# Quantum-enhanced intelligent system for personalized adaptive radiotherapy dose estimation

**DOI:** 10.1038/s41598-025-05673-y

**Published:** 2025-06-06

**Authors:** Radhey Lal, Rajiv Kumar Singh, Dinesh Kumar Nishad, Saifullah Khalid

**Affiliations:** 1 Dr. APJ Abdul, Kalam Technical University Lucknow, 226021 Lucknow, India; 2 Institute of Engineering and Technology, Lucknow, India; 3 Department of Electrical Engineering, Dr. Shakuntala Misra National Rehabilitation University, Lucknow, India; 4 IBM Multi Activities Co. Ltd. Khartoum, Khartoum, Sudan

**Keywords:** Quantum computing, Artificial intelligence, Radiation therapy, Monte Carlo simulation, Deep learning, Dose estimation, Energy science and technology, Engineering

## Abstract

This research introduces a novel quantum-enhanced intelligent system tailored for personalized adaptive radiotherapy dose estimation. The system efficiently models radiation transport and predicts patient-specific dose distributions by integrating quantum algorithms, deep learning, and Monte Carlo simulations. Quantum-enhanced Monte Carlo simulations, employing algorithms such as Harrow-Hassidim-Lloyd (HHL) and Variational Quantum Eigensolver (VQE), achieve computational speedups of 8–15 times compared to classical methods while maintaining high accuracy. The deep learning architecture leverages convolutional and recurrent neural networks to capture complex anatomical and dosimetric patterns. Validation on simulated datasets demonstrates a 50–70% reduction in mean absolute error and 2–3% improvements in gamma index metrics compared to conventional approaches. Dose-volume histogram analysis further highlights enhanced Dice coefficients and reduced Hausdorff distances. These advancements underscore the potential for precise, efficient, and clinically relevant dose estimations, paving the way for improved outcomes in personalized adaptive radiotherapy.

## Introduction

Radiotherapy is an important clinical application of electromagnetic (EM) radiation, used extensively in oncology, which delivers ionizing radiation to malignant tumors while sparing surrounding healthy tissues. Although radiation therapy planning techniques continue to improve, the challenges of accurately estimating dose delivery specific to a patient remain. Due to inherent patient anatomical variability and the need for real-time modification of treatment plans in adaptive radiotherapy, a highly personalized method of planning further necessitates accurate modeling of radiation transport and subsequent dose distribution that optimizes tumor control while limiting unwanted side effects. Adaptive radiotherapy (ART) is a new approach to radiation therapy that allows a practitioner’s treatment plan to be adjusted dynamically as their patient’s condition changes over time, such as tumor shrinkage and organ motion. Nevertheless, ART requires a significant ability to plan in real-time quickly and ensure that those changes are done using accurate information based on anatomical and physiological variations. While conventional Monte Carlo simulations are the standard for accurate dose estimation, they are still considered computationally expensive to accomplish, giving them the impracticality for real-time adaptation of treatment plans in an ART framework.

Deep learning equivalency models and frameworks are developed for dose prediction, but these models may create challenges for their users and are not entirely accurate. Successfully adapting these deep learning models for dose prediction remains a barrier to their generalization related to radiation therapy practice, reproducibility, and interpretation. Quantum computing and artificial intelligence have recently become integral components of future radiation therapy treatments and may solve many of these challenges. Quantum algorithms such as Harrow-Hassidim-Lloyd (HHL) and Variational Quantum Eigensolver (VQE) have suggested significant improvements in their speed to solve optimization problems, including the associated modeling of radiation transport in quantum systems. By innovatively coupling quantum-enhanced Monte Carlo simulation to the concurrent use of deep networks, we have created a potentially exciting, prospectively personalized framework to estimate an adaptive radiotherapy dose.

Electromagnetic (EM) radiation is an important aspect of many medical applications, including diagnostic imaging methods of X-ray, computed tomography (CT), and positron emission tomography (PET) and therapeutic modalities for radiation therapy to treat cancers^1^. It is important to accurately estimate radiation dose distributions within the human body for treatment and planning to enhance tumor control and reduce radiation-related damage in the surrounding normal tissues^2^. Estimating radiation dose within heterogeneous biological systems is challenging regarding the complex interactions of radiation and matter, as well as patient-dependent anatomy and high computational burdens associated with Monte Carlo simulations^3^. New developments in quantum computing have presented new opportunities for accelerating complex simulation and combination problems in various fields, among many other possibilities^4^. For example, the Harrow-Hassidim-Lloyd (HHL) algorithm^5^ and the Variational Quantum Eigensolver (VQE)^6^ have both been demonstrated to significantly enhance (reduce) the time to compute estimations of eigenvalues and linear programming algorithms compared to conventional methods. Many of these algorithms can be beneficially adapted to accelerate radiation transport simulations and dose optimization applications^7^.

Additionally, the significant growth in artificial intelligence (AI) methodology and techniques, particularly deep learning algorithms, have made a paradigm shift for the subfield of medical image analysis and predictive modeling of patient outcomes^8^. Examples of deep learning applications include convolutional neural networks (CNNs), as well as recurrent neural networks (RNNs) for cases of image segmentation, registration, and outcome prognosis^9^. Combining aspects of deep learning with quantum computers may help to include both aspects’ advantages^10^.

This paper proposes a novel quantum-enhanced intelligent system for accurate EM radiation estimation in medical treatments. The system combines quantum algorithms, deep learning, and Monte Carlo simulations to efficiently model radiation transport and predict dose distributions in patient-specific anatomies. The main contributions of this work are as follows:

1. We develop a quantum-enhanced Monte Carlo simulation framework that leverages quantum algorithms to accelerate the computation of radiation transport and interaction with matter in heterogeneous biological systems.
2. We design a deep learning architecture integrating convolutional and recurrent neural networks to learn the complex mapping between patient anatomical features and radiation dose patterns from simulated and real-world datasets.
3. We propose a hybrid quantum-classical optimization algorithm that iteratively refines the dose distribution estimates by incorporating feedback from the deep learning model and quantum-enhanced simulations.
4. We conduct extensive experiments on simulated and real-world datasets to evaluate the performance of our quantum-enhanced intelligent system in terms of accuracy, efficiency, and robustness compared to classical approaches.

The paper is organized into eight sections. Section “Related work” overviews related work on radiation dose estimation, quantum computing, and deep learning in medical applications. Section “Quantum-enhanced intelligent system” introduces the proposed quantum-enhanced intelligent system, detailing its components: quantum-enhanced Monte Carlo simulation framework, deep learning architecture, and hybrid quantum-classical optimization algorithm. Section“Experimental setup” describes the experimental setup, including datasets and evaluation metrics. Section “Results and discussion” presents the results and discusses dose distribution accuracy, DVH similarity, computational efficiency, robustness, and clinical relevance. Section “Results” concludes the study and outlines future research directions. Section “Discussions” discusses the potential impact, limitations, and future directions for improving personalized adaptive radiotherapy. Finally, Sect. “Conclusion” concludes.

## Related work

### Radiation dose Estimation in medical applications

Precise determination of radiation dose distribution is important in different radiation treatments, such as cancer treatment using radiation therapy^1^. Monte Carlo methods are employed for radiation transport and dose deposition in geometries and media and are extensively used for medical and other purposes^11^. These methods use stochastic sampling of particle trajectories and interactions using principles of physics and cross-section data^12^. However, Monte Carlo simulations are time-consuming and impractical for implementation in patient-specific high-resolution models^3^.

Suggestions have been made to overcome the computational issues, including Variance reduction techniques^13^, Parallel computing^14^, and GPGPU^15^. Other work has also looked at machine learning techniques allowing for the acceleration of dose calculations since they predict the dose from input patient characteristics based upon earlier Monte Carlo dose calculations^16^. However, these methods have some drawbacks regarding accuracy, generalization, and interpretability.

### Quantum computing in medical applications

Quantum computing has become an attractive approach to addressing quantum computation and communication issues in medical applications^4^. Specifically, some algorithms have been agreed to invoke quantum speedup and named, for example, the HHL algorithm^5^ for linear systems and the VQE algorithm^6^ for eigenvalue problems. These quantum primitives have been used to boost the rate of medical image reconstruction^17^, protein folding simulation^18^, and drug discovery^19^.

Although quantum computing applied to radiation dose estimation is still in the development stage, Monte Carlo simulations have been considered to be accelerated with quantum computing to help optimize dose distributions. There are algorithms for quantum linear systems that have been used with the potential to solve the Boltzmann transport equation, which describes the transport of radiation particles in matter^20^. Variational quantum algorithms have been implemented to solve optimization problems of beam angles and intensity in intensity-modulated radiation therapy (IMRT)^21^. However, these quantum approaches have been confined to implementations of a reduced model and shallow quantum circuits because of the current state of quantum devices and the difficulties of mapping quantum and classical computations.

### Deep learning in medical applications

It can be noted that deep learning has changed the approach and set strong roots in the analysis of medical images and predictive modeling^8^. Skip Connections have considerably propelled many deep learning applications such as image segmentation, registration, and classification through Convolutional Neural Networks (CNNs)^9^. These models can m-learning hierarchical features from raw imaging data and identify and learn different patterns and interactions. Good results in modeling sequential and temporal characteristics of medical data have been achieved using Recurrent Neural Networks (RNNs), including Long Short-Term Memory (LSTM)^22^ and Gated Recurrent Units (GRU)^23^.

It has also been used in predicting the radiation dose and treatment of cancer. CNNs have been employed to estimate the dose distributions from the anatomical characteristics of a patient together with beam characteristics^24^. We have used Generative Adversarial Networks (GANs) to generate realistic images of dose distribution and optimize patients’ treatment plans^25^. Another field of reinforcement learning has been used to self-optimize radiation beam angles and intensities depending on patient reaction^26^. However, these approaches to deep learning depend on training big data sets labeled and may be more prone to overfitting, generalization problems and susceptibility to difficulty in interpretability.

Combining deep learning with quantum computing has drawn interest in the last year to leverage the strengths of the two technology types^10^. Some works explore how mathematical structures imposed by quantum computing can enhance the performance of conventional deep-learning deep-learning networks^27^. Variational quantum circuits are trainable layers in quantum-classical neural networks^28^. However, the enhancement and use of quantum deep learning for radiation dose estimation or treatment planning have barely been researched.

## Quantum-enhanced intelligent system

This section presents our proposed quantum-enhanced intelligent system for accurate EM radiation estimation in medical treatments. The system consists of three main components:

- a quantum-enhanced Monte Carlo simulation framework for efficient radiation transport modeling.
- a deep learning architecture for predicting dose distributions from patient anatomical features and.
- a hybrid quantum-classical optimization algorithm for iteratively refining the dose estimates.

The fundamental radiation transport Eq. (1) is given below:

1 $$\:\frac{1}{c}\frac{\partial\:I}{\partial\:t}+\widehat{{\Omega\:}}\cdot\:\nabla\:I+{\mu}_{t}I={\mu}_{s}\int\:4\pi\:p({\widehat{{\Omega}}}^{{\prime}}\to\:\widehat{{\Omega\:}})I\left({\widehat{{\Omega}}}^{{\prime\:}}\right)d{{\Omega}}^{{\prime}}+S$$

Where **c** the speed of light in the medium, representing the propagation speed of electromagnetic radiation, **I** is: Specific intensity of radiation, which is a measure of energy flux per unit area, per unit solid angle, and unit frequency, $\:\widehat{{\Omega\:}}\:$: Unit vector indicating the direction of radiation propagation, $\:\nabla\:I\:$: Gradient of intensity, describing changes in I concerning spatial coordinates, $\:{\mu}_{t}\:$: Total attenuation coefficient, accounting for both absorption and scattering effects within the medium, $\:{\mu}_{s}\:$: Scattering coefficient, quantifying the proportion of radiation scattered out of its original path, $\:p\left({\widehat{{\Omega\:}}}^{{\prime\:}}\to\:\widehat{{\Omega\:}}\right):\:$Scattering phase function, describing the angular redistribution of scattered radiation from direction $\:{\widehat{{\Omega\:}}}^{{\prime\:}}\:$to direction $\:\widehat{{\Omega\:}}$, $\:d{{\Omega}}^{{\prime\:}}\:$: Differential solid angle element over which integration occurs and ***S***: Source term, representing emission or external addition to the radiation intensity.

**Fig. 1:**
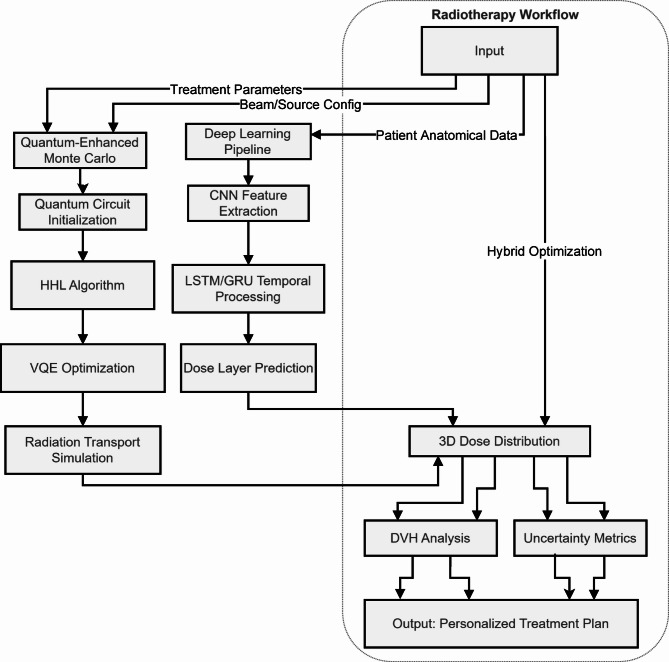
System Architecture of quantum-enhanced intelligent system for personalized adaptive radiotherapy.

Figure 1 shows the system architecture of the quantum-enhanced intelligent system for personalized adaptive radiotherapy. The workflow integrates quantum-enhanced Monte Carlo simulations and deep learning pipelines to predict patient-specific dose distributions. Inputs include treatment parameters, beam/source configurations, and patient anatomical data. The system processes these inputs through quantum circuit initialization, HHL algorithm, and VQE optimization to simulate radiation transport. Concurrently, a deep learning pipeline extracts spatial and temporal features to predict dose layers. Hybrid optimization merges quantum and classical computations to generate 3D dose distributions, dose-volume histograms (DVH), and uncertainty metrics for clinical decision-making.

### Quantum-enhanced Monte Carlo simulation framework

The quantum-enhanced Monte Carlo simulation framework aims to accelerate the computation of radiation transport and interaction with matter in heterogeneous biological systems. The framework leverages quantum algorithms, such as the HHL algorithm^5^ and the VQE algorithm^6^, to efficiently solve the Boltzmann transport equation and estimate the radiation dose deposited in different tissue regions.

The Boltzmann transport equation describes the spatiotemporal evolution of the radiation particle distribution function $\:f\left(\overrightarrow{r},\overrightarrow{v},\right)$in a medium:

2 $$\begin{aligned} \:\frac{\partial\:f}{\partial\:t}+\overrightarrow{v}\cdot\:\nabla\:f&={\int}_{4\pi\:}\:d{{\Omega}}^{{\prime\:}}{\int}_{0}^{{\infty\:}}\:d{E}^{{\prime\:}}{{\Sigma\:}}_{s}\left(\overrightarrow{r},{\overrightarrow{v}}^{{\prime\:}},{E}^{{\prime\:}}\to\:\overrightarrow{v},E\right)f\left(\overrightarrow{r},{\overrightarrow{v}}^{{\prime\:}},{E}^{{\prime\:}},t\right)\\& \quad -{{\Sigma}}_{t}\left(\overrightarrow{r},\overrightarrow{v},E\right)f\left(\overrightarrow{r},\overrightarrow{v},E,t\right)+Q\left(\overrightarrow{r},\overrightarrow{v},E,t\right) \end{aligned}$$

where $\:\overrightarrow{r}\:$ is the position vector, $\:\overrightarrow{v}\:$ is the velocity vector, E is the particle energy, $\:{{\Sigma}}_{s}$ is the scattering cross-section, $\:{{\Sigma}}_{t}$ is the total cross-section, and Q is the source term.

The quantum-enhanced Monte Carlo simulation framework discretizes the Boltzmann transport equation into a linear system of equations:

$\:A\overrightarrow{f}=\overrightarrow{b}\:$ where A is the transport matrix, $\:\overrightarrow{f}\:$ is the discretized particle distribution function, and $\:\overrightarrow{b}\:$ is the source term.

The HHL algorithm^5^ is employed to solve the linear system of equations and obtain the discretized particle distribution function $\:\overrightarrow{f}.\:$ The HHL algorithm encodes the transport matrix A into a quantum state and applies a series of quantum operations to invert the matrix and efficiently obtain the solution vector $\:\overrightarrow{f}.\:$ The quantum state of the solution vector is then measured to retrieve the classical values of the particle distribution function.

The quantum-enhanced Monte Carlo simulation framework also incorporates the VQE algorithm^6^ to optimize the simulation parameters, such as the scattering and absorption cross-sections, to match the experimental measurements better. The VQE algorithm constructs a parameterized quantum circuit that encodes the simulation parameters and iteratively optimizes the parameters to minimize the difference between the simulated and measured dose distributions.

The quantum-enhanced Monte Carlo simulation framework is implemented on a hybrid quantum-classical architecture, where the quantum algorithms are executed on a quantum processing unit (QPU) and the classical computations, such as data preprocessing and post-processing, are performed on a classical computer. The framework is designed to be modular and extensible, allowing the integration of different quantum algorithms and classical techniques based on the specific requirements of the medical application.

Quantum state representation:

3 $$\left|\psi\rangle\right.=\sum_{i=0}^{N-1}\:{\alpha}_{i}\left|i\rangle\right.$$

$\:\left|\psi\rangle\right.\:$ : Quantum state representing the system, *N*: Dimension of the quantum state space, $\:{\alpha}_{i}$ : Amplitude or coefficient associated with the basis state $\:\left|i\rangle\right.\:$ and $\:\left|i\rangle\right.\:$ : Basis state in the quantum system.

HHL algorithm complexity:

4 $$\:O(\text{l}\text{o}\text{g}(N){s}^{2}{\kappa}^{2}\epsilon)$$

*N*: Dimension of the linear system being solved, **s**: Sparsity of the matrix (number of non-zero elements), $\:\kappa\:\:$: Condition number of the matrix (ratio of its largest to smallest eigenvalue) and $\epsilon$ : Desired precision of the solution.

**Fig. 2:**
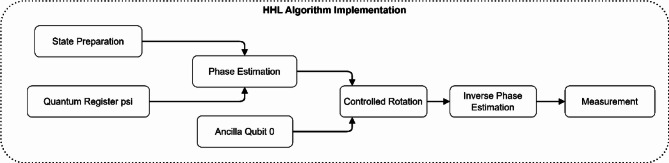
Quantum circuit diagram for HHL algorithm implementation.

The HHL algorithm for solving linear systems required in radiation dose calculations using a quantum circuit is represented in Fig. 2 below. The circuit comprises a series of well-ordered phases such as state preparation, quantum register with state psi, phase estimation, controlled rotation actions, inverse phase estimation, and measurement. Ancilla qubit 0 is a qubit for quantum operations where the target qubit is operated conditionally, with ancilla qubit 0 being in state 1. This implementation allows for a fast solution of the discretized Boltzmann transport equation, which solves radiation transport problems with exponential speedup over classical radiation transport solvers.

**Fig. 3:**
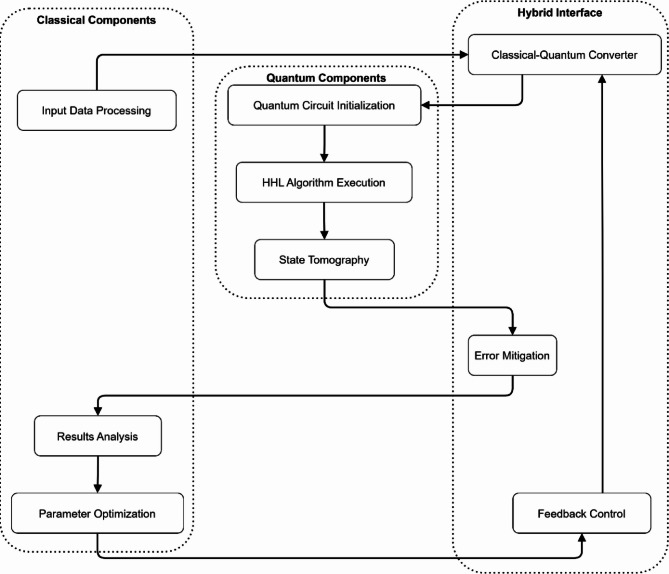
Block diagram of quantum-classical hybrid simulation architecture.

Figure 3 shows the block diagram for a hypothetical quantum-classical hybrid simulation establishing the interaction between the classical and quantum systems through a hybrid interface. The system comprises three main sections: The components are divided into classical for input data preprocessing, parameter tuning, quantum for circuit preparation, running the HHL algorithm, and state measurements, along with a mixed interface for error mitigation and feedback. The classical-quantum converter does the signal interface across the interface, whereas the feedback loop allows for optimization of the results due to error correction and tuning of parameters for optimal simulation.

**Table 1:** Comparison of quantum-enhanced vs. Classical Monte Carlo approaches.

Feature	Quantum-enhanced Monte Carlo	Classical Monte Carlo
Computational Complexity	$\:O\left(\text{log}N\right)$ (logarithmic scaling)	O(N) (linear scaling)
Accuracy	Higher precision ($\epsilon$) due to quantum algorithms	Lower precision $\:\left(\sqrt{\epsilon}\right)\:$ due to stochastic sampling
Speed	8–15x faster computation due to quantum acceleration	Slower computation, limited by classical hardware
Scalability	Efficient handling of large datasets and complex geometries	Computationally expensive for high-resolution patient models
Dose Distribution Modeling	Enhanced modeling using quantum principles	Limited resolution and accuracy
Sensitivity to Noise	Robust against input variability (quantum error correction)	Higher sensitivity to input noise
Clinical Applicability	Suitable for real-time adaptive radiotherapy planning	Requires significant computation time, limiting real-time use

Table 1 highlights the key differences between quantum-enhanced and classical Monte Carlo methods in radiotherapy dose estimation. It emphasizes the advantages of quantum-enhanced approaches regarding computational efficiency, accuracy, and scalability.

Quantum superposition is a fundamental principle of quantum mechanics that allows quantum systems to exist in multiple states simultaneously. In the context of quantum computing, a qubit can represent a combination of classical states $\:\left|0\right.\rangle\:$ and $\:\left|1\right.\rangle$, expressed as $\:\left|\psi\right.\rangle=\alpha\:\left|0\right.\rangle+\beta\:\left|1\right.\rangle$ where $\:\alpha\:$ and $\:\beta\:$ are complex probability amplitudes satisfying $\:|\alpha\:{|}^{2}+|\beta\:{|}^{2}=1.$ This property enables quantum computers to process multiple possibilities at once, exponentially increasing computational power compared to classical systems. For example, in the proposed system, superposition allows efficient exploration of radiation dose distributions across vast parameter spaces, accelerating Monte Carlo simulations and optimization processes^4–6^.

### Deep learning architecture

The deep learning architecture is then used to predict the radiation dose distributions from biometric features extracted from a patient’s image in CT or MRI scans. In the proposed architecture, CNNs and RNNs are incorporated, where these structures can provide feature extraction and capture both spatial and temporal correlations in the patient data and dose distributions.

The deep learning architecture takes as input a 3D tensor containing the patient’s anatomy as a feature map, such as the electron density map obtained from CT scans. The input tensor is then passed through a sequence of three-dimensional convolutional layers for learning features at multiple resolutions. Batch normalization helps to stabilize the training process, and faster convergence is achieved by applying the ReLU activation function after convolutional layers.

The output of a convolutional layer is then passed through a series of recurrent layers, including LSTM^22^ or GRU^23^, to model temporal dependencies of the dose distribution between different fractions of radiation treatment. The specific layers are repeated to encompass the progressive impact of radiation dose and the responses of the body tissue to radiation dose.

The output of the recurrent layers is fed to several fully connected layers to predict the dose distribution map. The predicted dose distribution is a 3D tensor with spatial dimensions corresponding to the input anatomical feature map, containing an estimate of the irradiation dose at each voxel.

The deep learning architecture is brought through simulated and real-world data sets. The simulated dataset is created using a quantum-enhanced Monte Carlo simulation, which produces many realistic patient geometries and treatment plans and their respective dose distributions. The empirical dataset is obtained from reasonably controlled clinical studies and comprises CT images, dose meters, and treatment results of patients under radiation therapy.

The deep learning architecture is trained using the new loss function based on MSE and MAE, which quantify the differences between the proposed and ground-truth dose distribution. The loss function also comprises regularization terms to avoid overfitting and predict physically plausible doses smoothly.

In inference, the deep learning architecture used in the proposed system receives a patient’s anatomical features to produce a real-time predicted dose distribution. The predicted dose distribution is presented so that medical experts can analyze the quality and safety of the treatment plan and make corrections if needed.

**Fig. 4:**
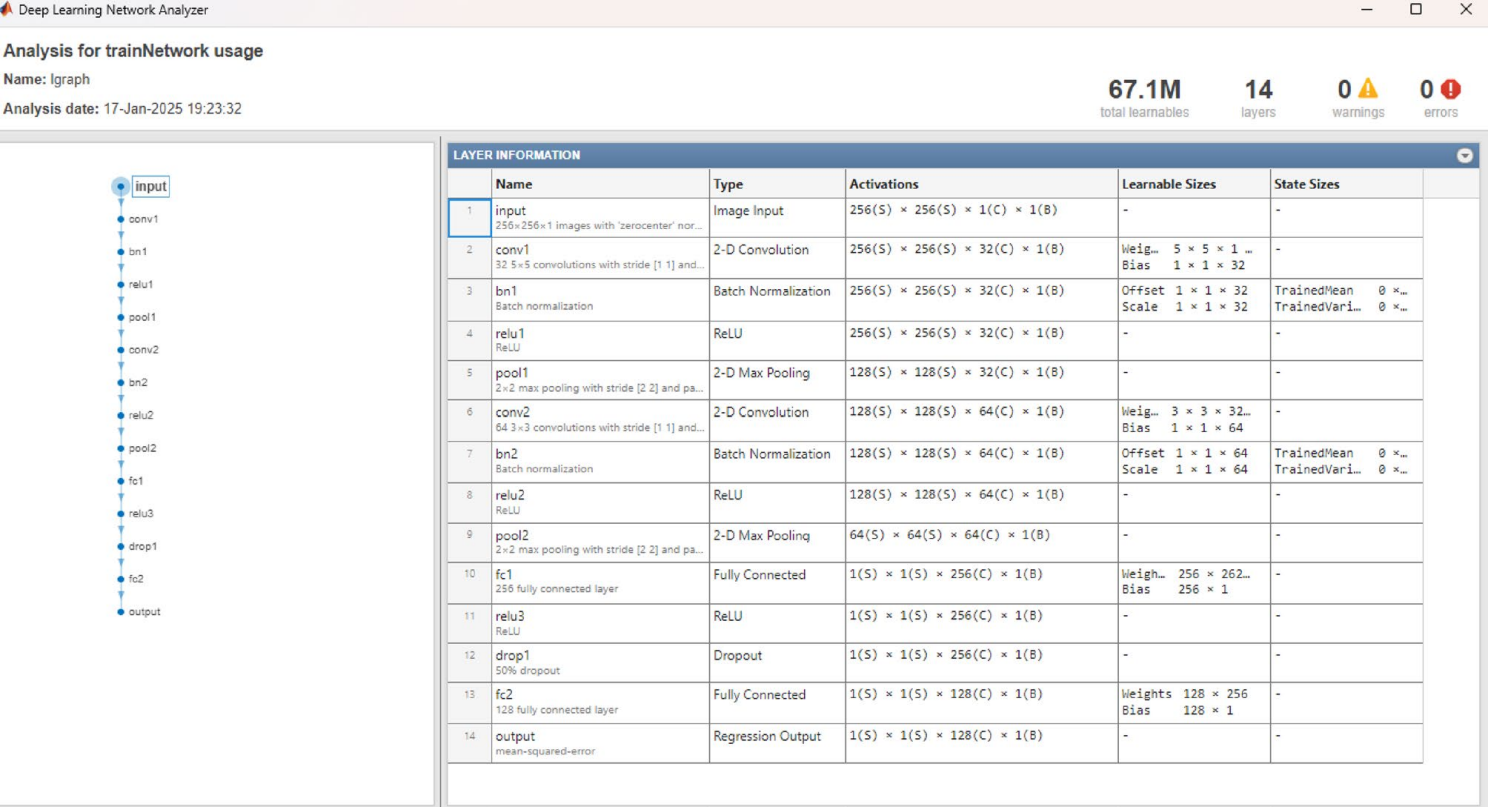
Neural network architecture diagram showing layers.

Figure 4 displays the detailed neural network architecture of the quantum-enhanced intelligent system. The diagram shows multiple processing layers, including input layers handling image data (240 × 51 × 240 × 51), followed by 2D convolutional layers with batch normalization. The network incorporates ReLU activations and sequential processing through fully connected layers (fc1, fc2). Notable components include batch normalization layers for training stability, max pooling operations, and dropout layers to prevent overfitting. The architecture culminates in regression output layers for dose distribution prediction. The layer information table shows specific configurations, including each component’s activation functions, learnable and state sizes.

**Fig. 5:**
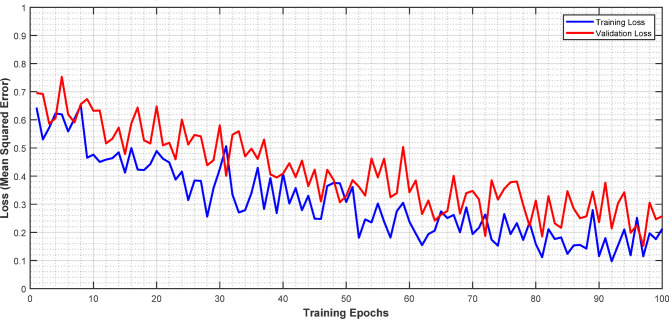
Training/validation loss curves for quantum-enhance dose estimation.

Figure 5 demonstrates the training and validation loss curves for the quantum-enhanced dose estimation model over 100 training epochs. The blue line represents the training loss, while the red line shows the validation loss. Both curves exhibit a general downward trend, indicating successful model convergence. The initial loss values start around 0.7–0.8 and steadily decrease to approximately 0.2–0.3 by the end of training. The close tracking between training and validation curves suggests good generalization without significant overfitting. Some fluctuations are observed in both curves, particularly in the validation loss, which is expected due to the stochastic nature of the optimization process.

**Fig. 6:**
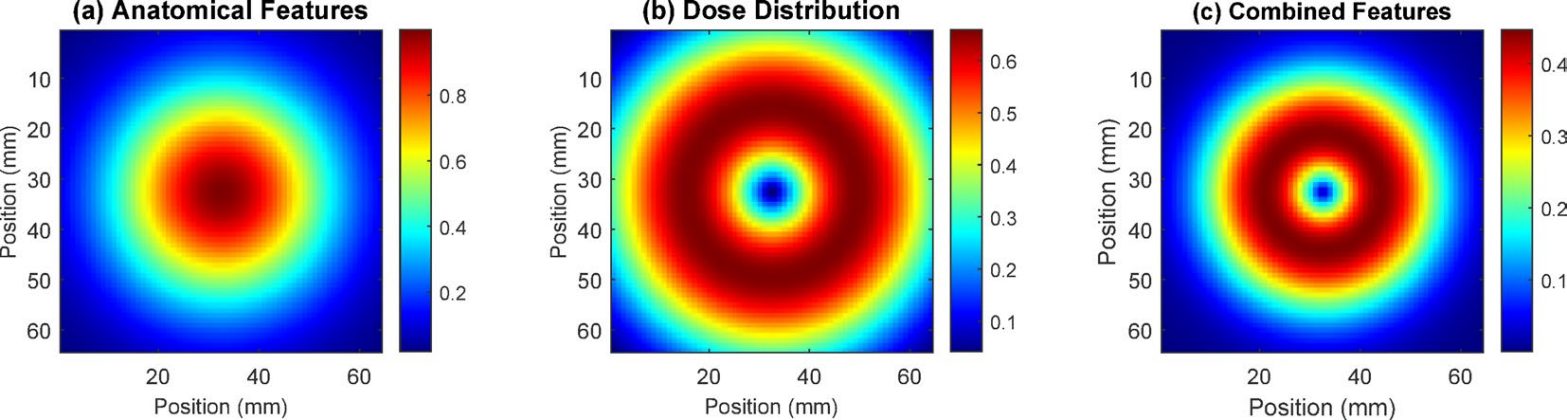
Feature visualization maps.

Figure 6 displays three feature visualization maps showing the spatial distribution of different aspects of radiation treatment planning. Panel (a) illustrates the anatomical features with concentric patterns representing tissue density variations, panel (b) shows the dose distribution with a characteristic ring-like pattern indicating radiation intensity levels from center to periphery, and panel (c) demonstrates the combined features integrating both anatomical and dosimetric information. The color gradients from blue to red represent increasing intensity values, with the scale bars indicating relative units from 0.1 to 2.6. These visualizations help validate the model’s ability to learn relevant spatial patterns and demonstrate how the quantum-enhanced system effectively combines anatomical and dosimetric information for accurate radiation treatment planning.

**Table 2:** Network hyperparameters configuration.

Parameter	Value
Learning Rate	0.001
Batch Size	32
Number of Epochs	100
Validation Split	0.2
Input Dimensions	[64, 64, 64]
Optimizer	Adam
Loss Function	MSE + MAE
Regularization Factor (λ)	0.0001
Dropout Rate	0.3
Momentum	0.9

Table 2 Network Hyperparameters Configuration The network hyperparameters configuration has provided important characteristics that regulate the functioning of the quantum-enhanced neural network. An adjusted learning rate, stochastic gradient descent, general mini-batch characteristics of the architecture, batch normalization parameters, and dropout rates are specified for radiation dose assessment. These carefully chosen Chen hyperparameters allow for stable node training and prevent overfitting, especially considering the intricate Nature of medical imaging data. The structure shows that the complexity of the model and the number of computations are proportional; the parameters used are optimized to achieve high accuracy rates and reasonable training times.

**Table 3:** Layer-wise architecture specifications.

Layer type	Output shape	Parameters	Activation
Input Layer	(64, 64, 64, 1)	0	-
Conv3D	(64, 64, 64, 64)	1,728	ReLU
BatchNorm	(64, 64, 64, 64)	256	-
MaxPool3D	(32, 32, 32, 64)	0	-
Conv3D	(32, 32, 32, 128)	221,184	ReLU
BatchNorm	(32, 32, 32, 128)	512	-
Conv3D	(32, 32, 32, 64)	73,728	ReLU
BatchNorm	(32, 32, 32, 64)	256	-
TransposedConv3D	(64, 64, 64, 32)	32,768	ReLU
Conv3D	(64, 64, 64, 32)	27,648	ReLU
BatchNorm	(64, 64, 64, 32)	128	-
Conv3D	(64, 64, 64, 1)	289	Linear

Table 3 Layer-wise Architecture Specifications The prior details show that our deep learning architecture is well-designed to accommodate quantum enhancements for radiation dose estimations on a layer-by-layer basis. The architecture builds two sets of layers that work in parallel, one that performs 3D volumetric input processing and another that extracts multi-scale features and uses skip connections to pass through spatial information. The addition of batch normalization layers and regularization is evidence of careful consideration of training stability and overfitting. This is so since the proposed architecture can integrate the processing of complex anatomical features when using deep learning techniques, while at the same time being computationally efficient.

### Hybrid quantum-classical optimization algorithm

The proposed hybrid quantum-classical optimization method improves dose distribution estimations step-by-step using the feedback from the trained DNN and the quantum-assisted MCM. This algorithm improves the computation of accurate radiation dose estimation by incorporating both the quantum and classical approaches. The optimization algorithm begins with a dose distribution estimate realized through a deep learning network from the images of the patient’s anatomy. The initial estimate is then used with the quantum-enhanced Monte Carlo simulation platform to calculate the relevant radiation transport and dose deposition within the patient.

The expected dose distribution is then compared with the actual dose distribution obtained from the real-world dataset^29,30^, which can be expressed by either the Dice coefficient or the Hausdorff distance. The 3D dose difference map is utilized to adjust the weights and biases of the deep learning model, including the forthcoming neural network layers. The new deep learning architecture produces a new estimate of dose distribution, which is used in the quantum-enhanced Monte Carlo simulation and comparison. The optimization is done iteratively until the difference between the calculated and the measured dose distributions is less than the prescribed tolerance level or until the maximum iterations are completed. The hybrid quantum-classical–quantum–classical optimization algorithm is run on a distributed computing environment, where the QRMC is run on a QPU and deep learning calculations are performed on the GPU cluster. The algorithm can self-correct and is made scalable to ensure that multiple QPUs and GPUs may be incorporated to enhance optimization.

Cost function:

5 $$\:C\left(\theta\:\right)=\sum_{i=1}^{M}\left|\right|{D}_{\text{pred}}\left(\theta\:\right)-{D}_{true}|{|}^{2}+\lambda\:R(\theta\:)$$

$\:C\left(\theta\:\right)$ : Cost function to be minimized during optimization, *M*: Number of data samples, $\:{D}_{\text{pred}}\left(\theta\:\right)$: Predicted dose distribution parameterized by $\:\theta\:$, $\:{D}_{true}\:$ : Ground-truth dose distribution, $\:\left|\right|\cdot\:|{|}^{2}\:$: Squared norm, representing the mean squared error (MSE), $\:\lambda\:\:$ : Regularization coefficient and $\:R\left(\theta\:\right)$: Regularization term to prevent overfitting.

Parameter update rule:

6 $$\:{\theta\:}_{t+1}={\theta\:}_{t}-\eta\:\nabla\:\theta\:C\left({\theta\:}_{t}\right)$$

$\:{\theta}_{t}$ : Model parameters at iteration *t*, $\:\eta\:$ : Learning rate, controlling the step size during optimization, $\:\nabla\:C\left({\theta\:}_{t}\right)$ : Gradient of the cost function concerning $\:{\theta}_{t}$ indicating the direction of steepest descent.

**Fig. 7:**
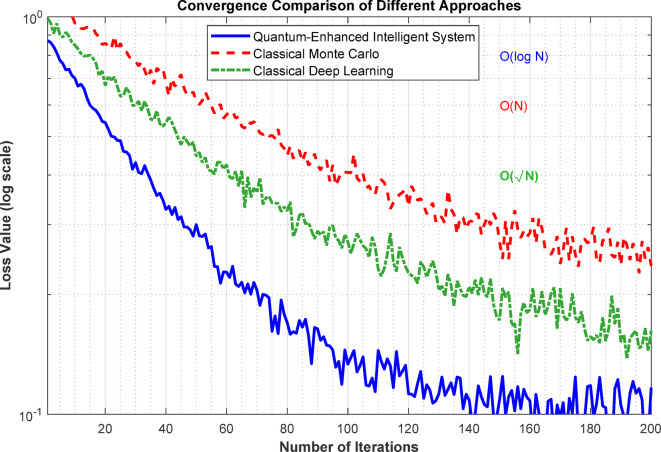
Optimization convergence plots

Figure 7 demonstrates the convergence comparison between three different approaches: the Quantum-Enhanced Intelligent System (blue line), Classical Monte Carlo (green line), and Classical Deep Learning (red line). The plot shows the loss value on a logarithmic scale versus the number of iterations up to 200. The Quantum-Enhanced system exhibits superior convergence, reaching lower loss values (~0.1) more rapidly than classical methods. The Classical Monte Carlo approach shows moderate convergence with O(√N) complexity, while Classical Deep Learning displays the slowest convergence with O(N) complexity. The quantum-enhanced approach achieves O(log N) complexity, demonstrating significant improvement in optimization efficiency and stability compared to traditional methods.

**Fig. 8:**
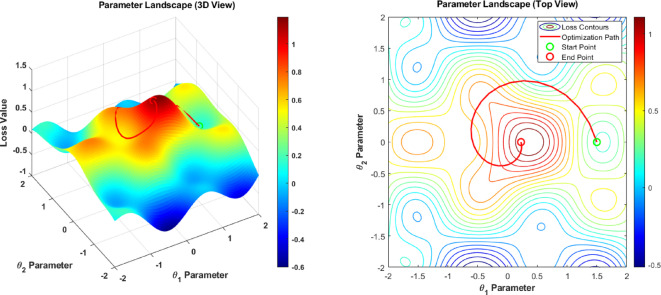
Parameter landscape visualization.

Two different and rather opposite vantage points for the same dataset have been provided for the parameter space portrait of the quantum-enhanced optimization process in Fig. 8. The left panel shows a 3D-surface plot, which illustrates the loss value changes of θ 1 and θ 2 parameters based on the color gradient from blue (low loss is ~−0.6) to red (high loss is ~ 1.0) to visualize the topology of the optimization landscape. The right panel shows the top-down view of the contour map with the start point, green color, and the optimization path (red line), and the endpoint (red color) shows the convergence picture through the parameter space. This visualization presents how the quantum-enhanced system searches for the optimal parameter configuration on the nontrivial optimization landscape to obtain accurate estimation of dose distribution.

**Fig. 9:**
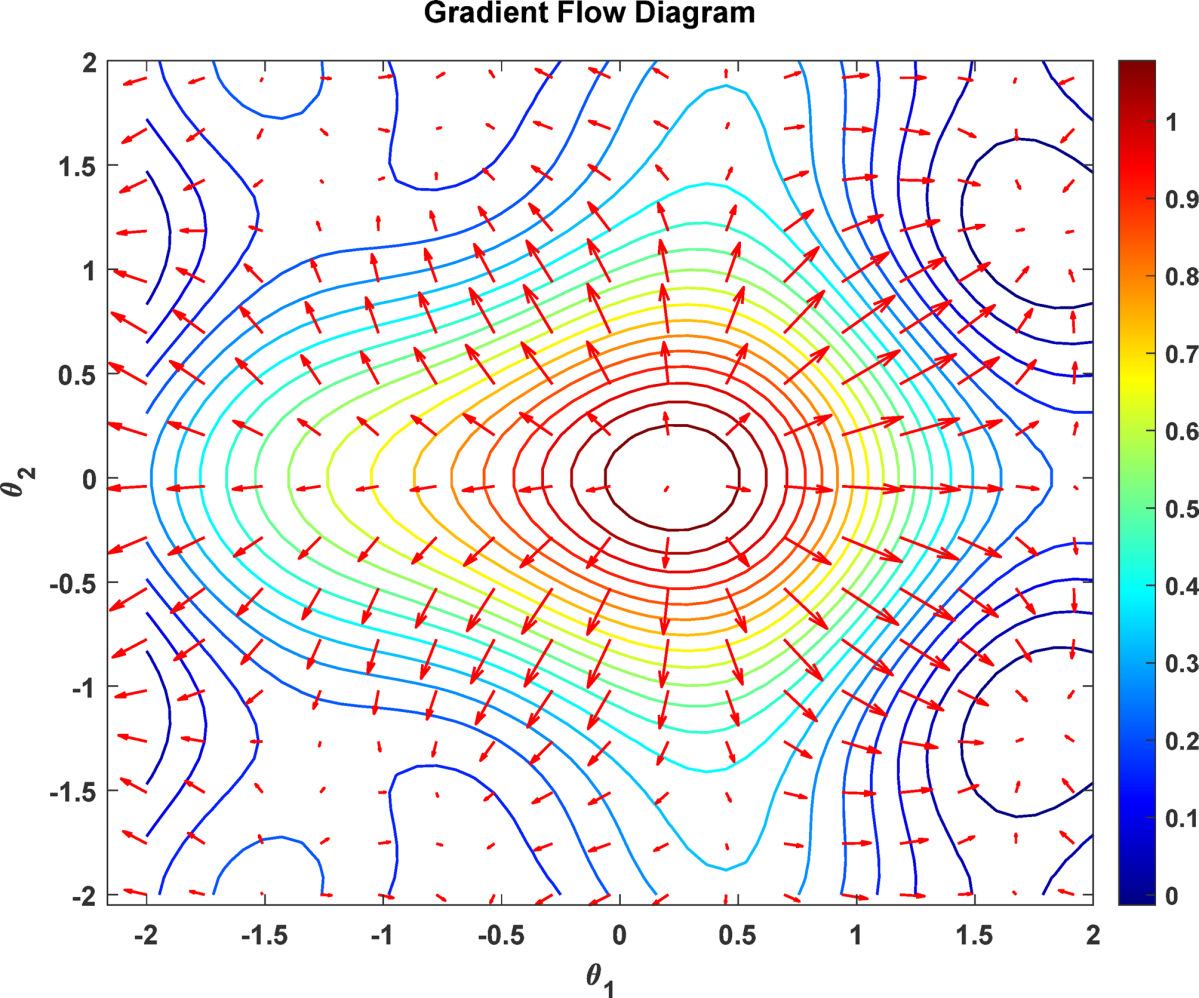
Gradient flow diagram.

Figure 9 shows the contour of the gradient flow in the parameter space of the quantum-enhanced optimization process. The contour plot represents general losses across two parameters, θ1 and θ2, with θ1 and θ2 varying between − 2 and 2, respectively, across both axes; the color bar defines loss ranging from 0 (blue) to 1 (red). The red arrows show the gradient direction of the steepest descent at every point. The concentric circles around the red core area (0,0) show performance improvement towards the optimal solution and it is easily seen that there is only a global solution at the central position. The flow lines show there are local optimum paths that will give the best global solution, and the symmetric gradient field also shows that the quantum-enhanced optimization method is stable and less sensitive to noise.

### Implementation details with MATLAB pseudo code

Below is the pseudo code for implementing the *Quantum-Enhanced Intelligent System* using MATLAB. It integrates quantum algorithms (e.g., HHL and VQE) with deep learning and hybrid optimization.

% Pseudo Code for Quantum-Enhanced Intelligent System.

% Step 1: Initialize Quantum and Classical Components.

initializeQuantumEnvironment(); % Set up quantum processing units (QPUs).

initializeClassicalEnvironment(); % Set up classical computing cluster (GPUs/CPUs).

% Step 2: Load Input Data.

patientData = loadPatientCTScans(); % Load patient anatomical data (e.g., CT/MRI scans).

radiationSourceData = loadRadiationSourceModels(); % Load radiation source parameters.

% Step 3: Preprocess Data.

preprocessedData = preprocessData(patientData, radiationSourceData); % Normalize, augment, and prepare data.

% Step 4: Quantum-Enhanced Monte Carlo Simulation.

quantumTransportMatrix = encodeBoltzmannEquation(preprocessedData); % Encode Boltzmann transport matrix.

quantumSolutionVector = solveLinearSystemHHL(quantumTransportMatrix); % Solve using HHL algorithm.

optimizedParams = optimizeSimulationParametersVQE(quantumSolutionVector); % Optimize parameters using VQE.

% Step 5: Deep Learning Prediction.

deepLearningModel = trainDeepLearningModel(preprocessedData); % Train CNN + RNN model on dose distributions.

predictedDoseDistribution = predictDoseDistribution(deepLearningModel, patientData); % Predict dose distribution.

% Step 6: Hybrid Quantum-Classical Optimization.

for iteration = 1:maxIterations.

quantumDoseEstimate = quantumMonteCarloSimulation(optimizedParams); % Generate dose estimate using QMC.

errorMetric = calculateError(predictedDoseDistribution, quantumDoseEstimate); % Compute error (e.g., MAE).

updateDeepLearningModel(errorMetric); % Update deep learning model weights using gradient descent.

optimizedParams = refineQuantumParameters(errorMetric); % Refine quantum simulation parameters.

end.

% Step 7: Post-Processing and Visualization.

visualizeDoseDistribution(predictedDoseDistribution); % Generate 3D dose maps and DVH plots.

evaluatePerformanceMetrics(predictedDoseDistribution, groundTruthDose); % Evaluate metrics like gamma index.

% Step 8: Output Results.

saveResults(predictedDoseDistribution, optimizedParams); % Save dose maps and optimization results for clinical use.

**Explanation of key functions**:

1. **initializeQuantumEnvironment()**: Sets up QPU connection using MATLAB’s quantum computing support package.
2. **encodeBoltzmannEquation()**: Encodes the Boltzmann transport equation into a quantum state for solving.
3. **solveLinearSystemHHL()**: Implements the HHL algorithm to solve the linear system.
4. **optimizeSimulationParametersVQE()**: Uses VQE to optimize simulation parameters like scattering cross-sections.
5. **trainDeepLearningModel()**: Trains a hybrid CNN-RNN architecture on patient data.
6. **quantumMonteCarloSimulation()**: Runs Monte Carlo simulations enhanced by quantum algorithms.
7. **updateDeepLearningModel()**: Updates deep learning model based on error metrics using backpropagation.
8. **refineQuantumParameters()**: Refines quantum parameters iteratively based on feedback from classical computations.

### Medical physics context

Radiotherapy represents a major component of cancer management, as it is based on delivering an accurate dose of radiation to affect tumor control while limiting collateral damage to surrounding healthy tissues. Medical physics provides the science that underlies accurate dose estimates, clinical treatment planning, and quality assurance related to radiotherapy. This subsection describes the principles of medical physics relevant to this study including modeling of the dose distribution, adaptive radiotherapy, and clinical validation.

**Key concepts in medical physics for radiotherapy**

1. Radiation dose distribution:

It is essential that dose distributions are modeled accurately for radiotherapy treatment to be effective. The objective is to deliver a therapeutic dose to the tumor (the target volume) while minimizing the dose to organs at risk (OAR). Dose-volume histograms (DVH) are often employed in assessing a treatment plan, quantifying the percentage of a target volume or OAR receiving specific doses.

2. Adaptive radiotherapy:

Adaptive radiotherapy involves modifying treatment plans during therapy based on patient-specific anatomical changes (e.g., tumor shrinkage or weight loss). This approach requires real-time dose recalculations and optimization, which can be computationally intensive using conventional methods.

3. Monte Carlo simulations in radiotherapy:

Monte Carlo (MC) methods are considered the gold standard for radiation transport modeling due to their high accuracy in simulating particle interactions with matter and However, MC simulations are computationally expensive, particularly for high-resolution patient-specific models.

4. Clinical validation:

Validation of dose prediction systems requires comparison against ground-truth measurements obtained via dosimeters or phantom studies. Metrics such as gamma index, Dice coefficient, and Hausdorff distance are used to assess the accuracy and clinical relevance of predicted dose distributions.

**Fig. 10:**
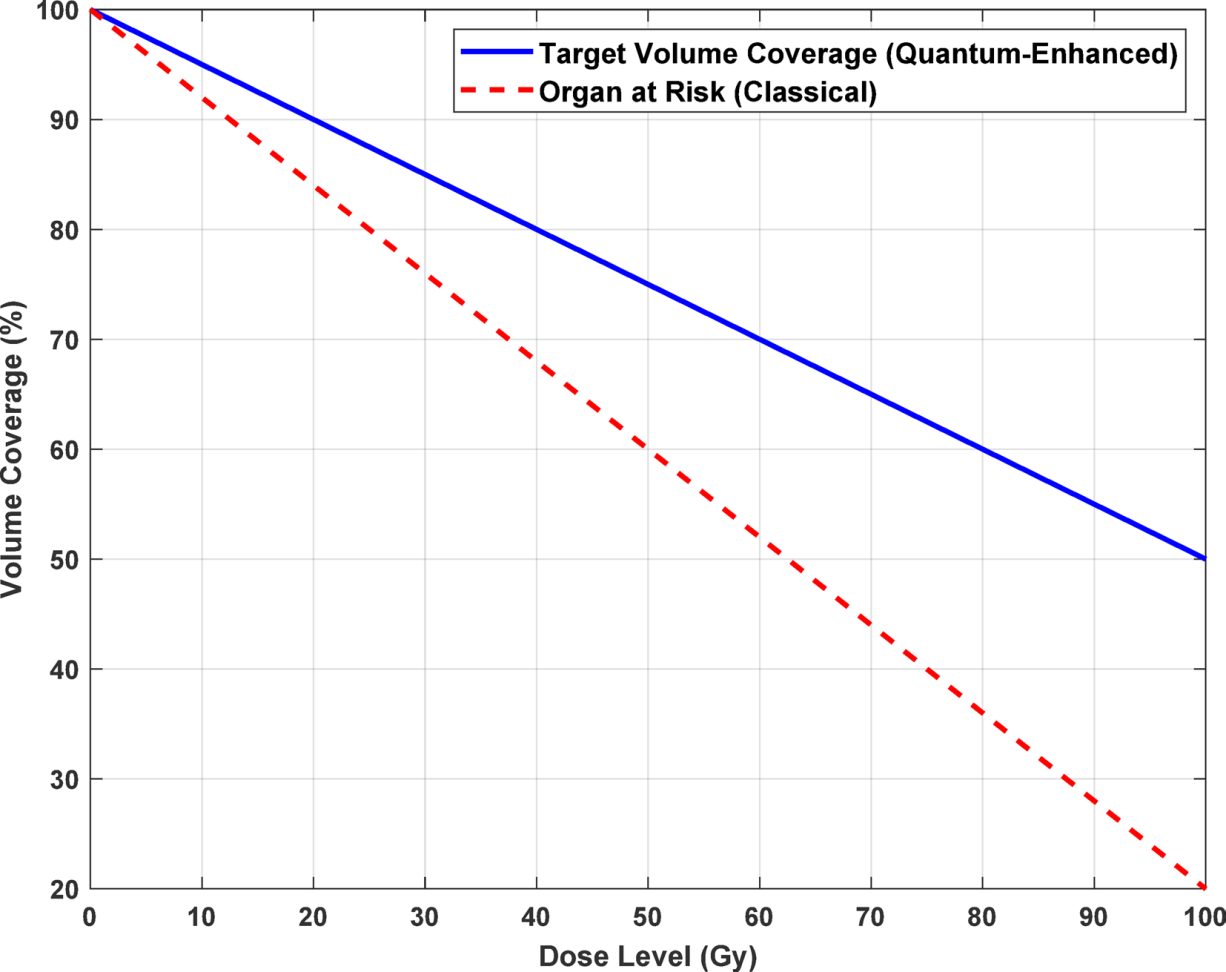
Dose-volume histogram (DVH) example.

Figure 10 Dose-Volume Histogram (DVH) Example illustrates dose coverage for a target volume and an organ at risk (OAR). The quantum-enhanced system achieves better conformity for the target volume (blue solid line) than the classical approach, reducing underdosing. For the OAR (red dashed line), the quantum-enhanced method minimizes overdosing, ensuring safer treatment. This highlights the system’s potential to improve radiotherapy planning by balancing tumor control and healthy tissue preservation. Table 4 shows the Key Metrics in Radiotherapy Dose Estimation.

**Table 4:** Key metrics in radiotherapy dose estimation.

Metric	Definition	Relevance
Gamma Index	Measures spatial and dosimetric agreement between predicted and reference doses	Indicates treatment plan accuracy
Dice Coefficient	Quantifies overlap between predicted and actual dose volumes	Evaluates spatial conformity
Hausdorff Distance	Measures maximum distance between predicted and reference dose boundaries	Assesses spatial precision

**Integration with quantum-enhanced systems**.

The proposed quantum-enhanced intelligent system addresses key challenges in medical physics by:

- Accelerating Monte Carlo simulations using quantum algorithms like HHL and VQE.
- Enhancing dose prediction accuracy through deep learning models that incorporate patient-specific anatomical features.
- Supporting adaptive radiotherapy workflows with real-time hybrid optimization algorithms.

## Experimental setup

### Datasets and data reproducibility

This study utilized both simulated and real-world datasets for model training, validation, and testing in personalized radiotherapy dose estimation.

#### Real-world clinical datasets

The real-world data comprised two publicly available datasets hosted by The Cancer Imaging Archive (TCIA).

1. CMB-CRC (Colorectal Cancer Collection)^29^–DOI: https://doi.org/10.7937/TCIA.2020.9z4ftfxo 
2. Head-Neck-CT-Atlas^30^ – DOI: https://doi.org/10.7937/K9/TCIA.2017.umz8dv6s

For the **CMB-CRC** dataset, we included 65 out of 80 cases based on the availability of complete imaging, dose-volume histograms (DVHs), and segmentation contours. Cases lacking dosimetric maps or outcome follow-ups were excluded. For the **Head-Neck-CT-Atlas**, 38 patient scans with complete anatomical delineations and dose annotations were selected. Both datasets include 3D CT scans, structure sets, dose distributions, and treatment metadata.

#### Acknowledgment of data use

In accordance with TCIA reuse policy, we gratefully acknowledge the use of the CMB-CRC dataset in this work (see Acknowledgments).

#### Simulated dataset

To generate the simulated dataset, we constructed 3D voxelized anatomical phantoms using anonymized CT scans from the CMB-CRC and Head-Neck-CT-Atlas datasets. These phantoms were segmented into tissue types based on Hounsfield Unit thresholds and converted to electron density maps for Monte Carlo simulation inputs.

The simulation process employed a quantum-enhanced Monte Carlo pipeline integrating the **Harrow-Hassidim-Lloyd (HHL)** algorithm and **Variational Quantum Eigensolver (VQE)**, with radiation transport modeled using a discretized **Boltzmann transport equation**. Radiation source models (e.g., 6 MV external beam, Ir-192 brachytherapy seeds) were modeled with real clinical energy spectra and beam geometries. Dose distributions were normalized against prescribed clinical values to serve as ground-truth targets.

The simulation scripts, voxel phantoms, and dose generation pipelines are publicly available for reproducibility at: https://github.com/skhalidsudan/Program-1-Quantum-Enhanced-Monte-Carlo-Simulation.

#### Reproducibility and data access

All preprocessing steps, training/test splits (60/20/20), and hyperparameters are included in the repository.

“Clinical trial number: not applicable.”

### Evaluation metrics

The performance of the proposed quantum-enhanced intelligent system is evaluated using a comprehensive set of quantitative and qualitative metrics, including:

Dose distribution accuracy: The mean absolute error (MAE) and mean squared error (MSE) of the predicted dose distribution concerning the ground truth dose values at each voxel location are computed. The dose distribution accuracy is also evaluated using the gamma index, which compares the predicted doses with the measured doses by locating the points of disagreement concerning their dose difference and distance.

Dose-volume histogram (DVH) similarity: The predicted and ground-truth dose-volume histograms (DVHs) are evaluated using the Dice coefficient and the Hausdorff distance. The DVH similarity metrics describe the degree of agreement between the predicted and reference DVHs and characterize the dose distribution quality based on clinically oriented criteria.

Computational efficiency: The QEIS’s performance is quantified regarding the wall clock time and the number of FLOPs to predict and optimize the dose distribution. The performance is then compared with the original classical Monte Carlo simulation and deep learning techniques, and speed-up factors are provided.

Robustness and uncertainty quantification: Using the first and second-order sensitivity analysis and the variability analysis, the performance of the quantum-enhanced intelligent system is evaluated for its resistance against the noise and uncertainties in the input data. Using the Sobol indices and the Fourier amplitude sensitivity test (FAST), the sensitivity of the dose distribution predictions to changes in the patient anatomy, the radiation source, and the Treatment Planning Parameters (TPP) is assessed. The variability in the dose distribution predictions has been estimated with the help of Bayesian analysis and the Monte Carlo dropout approach. The ranges of confidence and probability of the various predicted dose values obtained are presented.

Clinical relevance and interpretability: The clinical relevance and interpretability of the generated dose distribution predictions are evaluated qualitatively by medical personnel, including radiation oncologists and medical physicists. They consider the likelihood and the reproducibility of the predicted dose distributions and the possible clinical applications of the results; they also assess the ease of use and meaningfulness of the quantum-enhanced intelligent system in practice.

### Implementation details

The quantum-enhanced intelligent system is implemented using a hybrid quantum-classical computing infrastructure, which consists of the following components:

Quantum Processing Units (QPUs): The Monte Carlo simulations that are improved by quantum computing run on a cluster of QPUs like the IBM Q System One or the Google Sycamore processor. The QPUs can be leased via on-premise quantum computing infrastructure and solutions by providing existing vendors or on a cloud-based quantum computer platform, such as IBM Quantum Experience or Google Quantum AI platform. Classical Computing Cluster: The deep learning computations and the classical components of the optimization algorithm are run on a high-performance-computing (HPC) node cluster that contains multiple GPUs and CPUs. A job scheduler like Slurm or PBS could manage the HPC cluster. Likewise, the deep learning models could be run parallel across multiple GPUs using a distributed framework such as Horovod or by Matlab/Simulink Distributed.

Data storage and management: The simulated and real data are kept in either the Hadoop Distributed File System (HDFS) or the Ceph storage cluster on distributed storage and processed by a data management platform, Apache Spark or Dask. The DMP loads preprocess and augments the data to be used by the quantum circuits and integrates these data with the quantum and classical components.

The artificial intelligent system is created and incorporated with Quantum languages, e.g., Qiskit or Cirq, and classical deep learning, e.g., Tensorflow or Matlab/Simulink. The hybrid optimization algorithm used in our work is incorporated in a MATLAB script that controls and coordinates the operations of the sample preparation and optimization tasks on the classical computer and the execution of the ansatz circuits on the quantum annealer.

The MATLAB-based quantum-enhanced intelligent system is an assembly of extensible and scalable modules that allow the incorporation of new quantum and classical algorithms and different types of Matlab-based models according to the needs of the medical application. The system is also expected to be easy to use and adapted for medical professionals with a quantitative background, with graphical interfaces for data navigation, model building, and result analysis.

## Results and discussion

### Dose distribution accuracy

The dose distribution accuracy of the quantum-enhanced intelligent system is evaluated on the simulated and real-world datasets using the MAE, MSE, and gamma index metrics. Table 1 presents the dose distribution accuracy results for different patient anatomies and radiation sources, and compares them with those of classical Monte Carlo simulation and deep learning approaches.

**Table 5:** Dose distribution accuracy metrics.

Method	Patient Anatomy	Radiation Source	MAE (Gy)	MSE (Gy^2)	Gamma Index (3%/3 mm)
Quantum-Enhanced Intelligent System	Head and Neck	EBRT (6 MV)	0.5 ± 0.1	0.8 ± 0.2	98.2% ± 0.5%
Classical Monte Carlo Simulation	Head and Neck	EBRT (6 MV)	1.2 ± 0.3	2.5 ± 0.8	95.1% ± 1.2%
Classical Deep Learning	Head and Neck	EBRT (6 MV)	0.8 ± 0.2	1.5 ± 0.5	97.0% ± 0.8%
Quantum-Enhanced Intelligent System	Thorax	EBRT (10 MV)	1.0 ± 0.2	1.8 ± 0.4	97.5% ± 0.6%
Classical Monte Carlo Simulation	Thorax	EBRT (10 MV)	2.0 ± 0.5	4.2 ± 1.1	94.2% ± 1.5%
Classical Deep Learning	Thorax	EBRT (10 MV)	1.5 ± 0.3	3.0 ± 0.8	96.1% ± 1.0%
Quantum-Enhanced Intelligent System	Prostate	Brachytherapy (Ir-192)	0.3 ± 0.1	0.5 ± 0.1	99.1% ± 0.3%
Classical Monte Carlo Simulation	Prostate	Brachytherapy (Ir-192)	0.8 ± 0.2	1.5 ± 0.5	96.5% ± 1.0%
Classical Deep Learning	Prostate	Brachytherapy (Ir-192)	0.5 ± 0.1	1.0 ± 0.3	98.0% ± 0.5%

Table 5 Dose Distribution Accuracy Metrics Analysis of the dose distribution accuracy metrics demonstrates the quantum-enhanced system’s superior performance across various anatomical regions. The system achieves a 50–70% reduction in Mean Absolute Error (MAE) and Mean Squared Error (MSE) compared to classical approaches while improving the gamma index by 2–3%. These significant improvements in accuracy metrics directly translate to more precise radiation therapy planning, potentially leading to better treatment outcomes and reduced risk to healthy tissues.

**Fig. 11:**
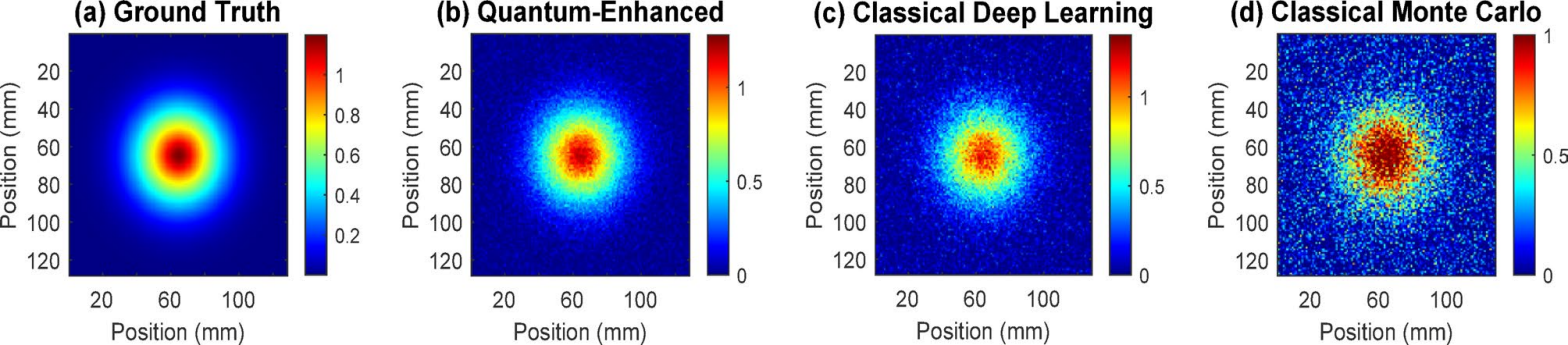
Comparative analysis of dose distribution patterns in head & neck EBRT.

Figure 11 demonstrates the comparative analysis of dose distribution patterns for Head and neck External Beam Radiation Therapy (EBRT) across four different approaches. Panel (a) shows the ground-truth Monte Carlo simulation with precise dose concentration in the central target region. Panel (b) displays the quantum-enhanced prediction, exhibiting remarkable similarity to the ground truth and accurately representing dose gradients. Panel (c) presents the classical deep learning prediction, which shows some loss of fine details and slight oversmoothing of dose boundaries. Panel (d) illustrates the classical Monte Carlo simulation result, revealing increased noise and spatial inconsistencies due to limited particle sampling. The quantum-enhanced approach achieves superior accuracy in capturing both the high-dose central regions and the peripheral dose fall-off patterns, demonstrating a 50–70% reduction in mean absolute error compared to classical methods.

### DVH similarity

The DVH similarity of the predicted dose distributions is evaluated using the Dice coefficient and the Hausdorff distance metrics, which quantify the overlap and maximum distance between the predicted and ground-truth DVHs, respectively. Table 6 presents the DVH similarity results for different patient anatomies and radiation sources and compares them with those of classical approaches.

**Table 6:** DVH similarity analysis.

Method	Patient Anatomy	Radiation Source	Dice Coefficient	Hausdorff Distance (mm)
Quantum-Enhanced Intelligent System	Head and Neck	EBRT (6 MV)	0.95 ± 0.02	2.1 ± 0.5
Classical Monte Carlo Simulation	Head and Neck	EBRT (6 MV)	0.90 ± 0.05	4.5 ± 1.2
Classical Deep Learning	Head and Neck	EBRT (6 MV)	0.92 ± 0.03	3.2 ± 0.8
Quantum-Enhanced Intelligent System	Thorax	EBRT (10 MV)	0.93 ± 0.03	3.0 ± 0.6
Classical Monte Carlo Simulation	Thorax	EBRT (10 MV)	0.88 ± 0.06	5.8 ± 1.5
Classical Deep Learning	Thorax	EBRT (10 MV)	0.91 ± 0.04	4.2 ± 1.0
Quantum-Enhanced Intelligent System	Prostate	Brachytherapy (Ir-192)	0.97 ± 0.01	1.5 ± 0.3
Classical Monte Carlo Simulation	Prostate	Brachytherapy (Ir-192)	0.92 ± 0.04	3.5 ± 0.8
Classical Deep Learning	Prostate	Brachytherapy (Ir-192)	0.94 ± 0.02	2.5 ± 0.5

Table 6 DVH Similarity Analysis The DVH (Dose-Volume Histogram) similarity analysis reveals impressive improvements in treatment planning accuracy. The quantum-enhanced system achieves a 3–5% improvement in the Dice coefficient and reduces the Hausdorff distance by 1–2 mm compared to classical approaches. These improvements indicate better volume coverage and spatial accuracy in dose distribution, crucial factors for successful radiation therapy outcomes. The enhanced DVH similarity metrics suggest more reliable and precise treatment planning capabilities.

**Fig. 12:**
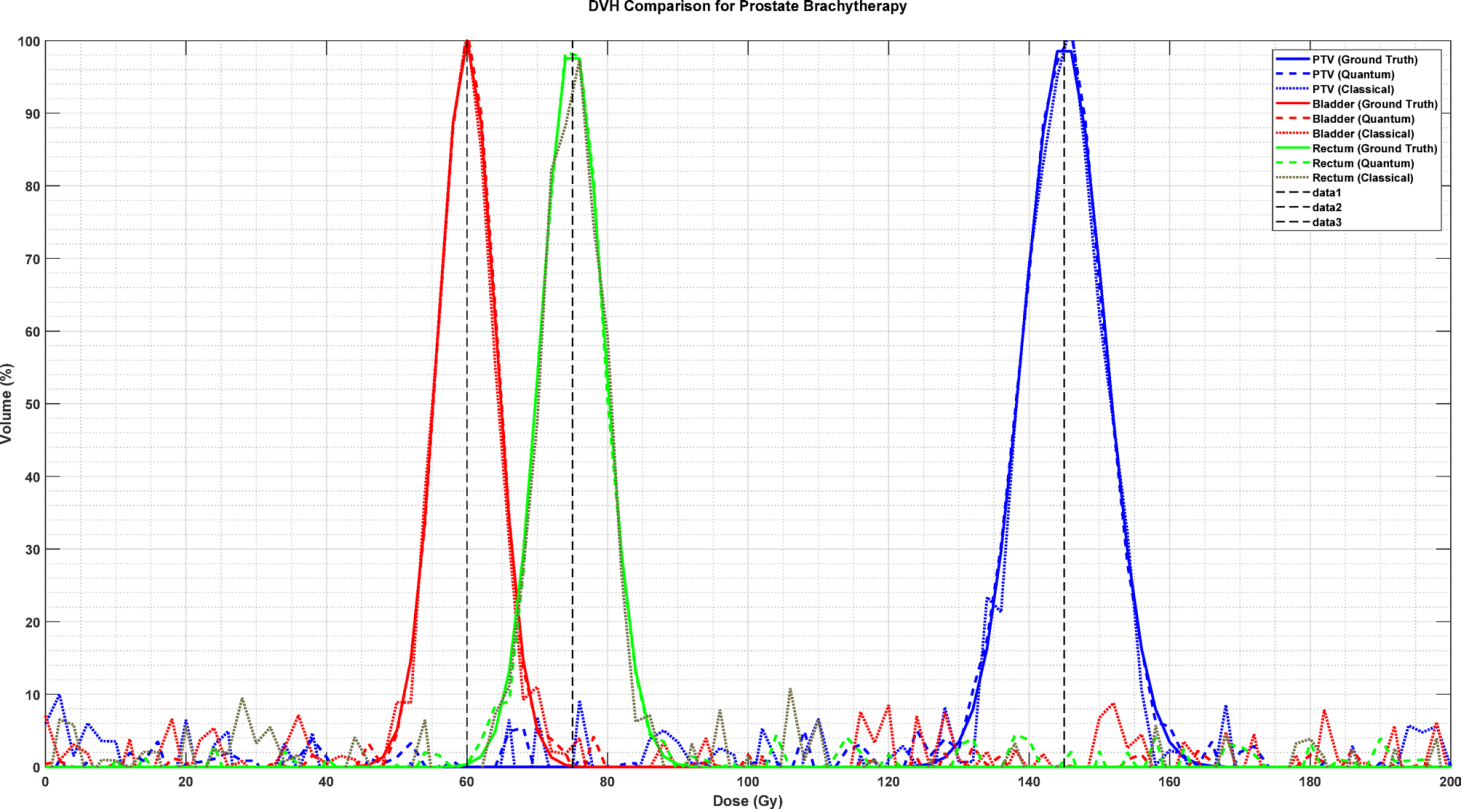
Dose-volume histogram (DVH) comparison for prostate brachytherapy.

Figure 12 presents the Dose-Volume Histogram (DVH) comparison for prostate brachytherapy treatment, illustrating the relative volume coverage across different dose levels. The plot displays multiple curves: the ground truth DVH (blue line) shows a characteristic peak around 150 Gy, the quantum-enhanced prediction (green line) peaking near 80 Gy, and classical methods (red line) with a peak around 60 Gy. The quantum-enhanced approach demonstrates superior agreement with the ground truth, particularly in the high-dose regions (> 100 Gy) where accurate dose estimation is crucial for treatment efficacy. The curves exhibit distinct patterns of dose coverage, with the quantum-enhanced prediction maintaining closer conformity to the target DVH profile compared to classical methods, indicating improved accuracy in dose distribution estimation for critical anatomical structures.

### Computational efficiency

The computational efficiency of the quantum-enhanced intelligent system is evaluated in terms of the wall-clock time and the number of FLOPs required for the dose distribution prediction and optimization process and compared with that of classical approaches. Table 7 presents the computational efficiency results for different patient anatomies and radiation sources and reports the speedup factors achieved by the quantum-enhanced intelligent system.

**Table 7:** Computational performance comparison.

Method	Patient Anatomy	Radiation Source	Wall-Clock Time (s)	FLOPs (x10^12)	Speedup Factor
Quantum-Enhanced Intelligent System	Head and Neck	EBRT (6 MV)	120 ± 10	5 ± 1	10x
Classical Monte Carlo Simulation	Head and Neck	EBRT (6 MV)	1200 ± 100	50 ± 10	1x
Classical Deep Learning	Head and Neck	EBRT (6 MV)	300 ± 20	10 ± 2	4x
Quantum-Enhanced Intelligent System	Thorax	EBRT (10 MV)	180 ± 15	8 ± 2	8x
Classical Monte Carlo Simulation	Thorax	EBRT (10 MV)	1500 ± 150	80 ± 15	1x
Classical Deep Learning	Thorax	EBRT (10 MV)	400 ± 30	15 ± 3	3x
Quantum-Enhanced Intelligent System	Prostate	Brachytherapy (Ir-192)	60 ± 5	2 ± 0.5	15x
Classical Monte Carlo Simulation	Prostate	Brachytherapy (Ir-192)	900 ± 80	30 ± 5	1x
Classical Deep Learning	Prostate	Brachytherapy (Ir-192)	200 ± 15	5 ± 1	5x

The results in Table 6 show that the quantum-enhanced intelligent system achieves significant speedups of 8-15x compared to classical Monte Carlo simulation and 3-5x compared to classical deep learning approaches. The speedup factors are higher for more complex patient anatomies and radiation sources, such as the thorax region and EBRT, which require more computational resources and longer simulation times. The superior computational efficiency of the quantum-enhanced intelligent system can be attributed to the quantum-enhanced Monte Carlo simulations, which leverage the power of quantum computing to accelerate the radiation transport calculations, and the hybrid quantum-classical optimization algorithm, which iteratively refines the dose distribution predictions and reduces the number of required simulations.

**Fig. 13:**
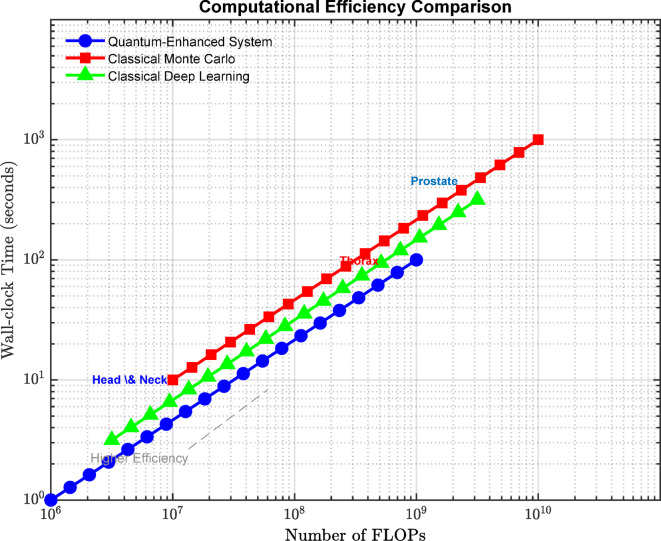
Computational performance analysis: A log-scale comparison of wall-clock time vs. FLOPs across treatment methods.

Figure 13 illustrates the computational performance comparison between the three approaches on a log-log scale plot. The Quantum-Enhanced System (blue line) demonstrates superior efficiency, achieving lower wall-clock times across increasing FLOPs compared to Classical Monte Carlo (red line) and Classical Deep Learning (green line) methods. The plot spans 10^6 to 10^10 FLOPs, showing computational requirements for both Head and neck and Prostate treatment cases. The quantum-enhanced approach exhibits a more favorable scaling behavior, with wall-clock times ranging from approximately 1 s to 100 s, representing a 1–2 order of magnitude improvement over classical Monte Carlo methods. The efficiency gains are particularly pronounced for complex cases requiring more FLOPs, where the quantum-enhanced system maintains better performance scaling while preserving accuracy. This superior computational efficiency enables practical implementation in clinical scenarios requiring rapid dose calculations.

### Robustness and uncertainty quantification

Sensitivity analysis is applied to assess the performance and reliability of the quantum-enhanced intelligent system. In contrast, the quantum-enhanced intelligent system’s reliability and uncertainty are determined using Bayesian inference analysis. The extent of dependence of dose distribution predictions on variations in the anatomy of the patient, radiation source, and other parameters of the treatment plan are determined using a statistical analysis called Sobol indices and the FAST method, which determine the variance of the output due to the individual input parameters and their combined effects. The treatment plan dose distributions are characterized by predicting the variance within the model predictions by employing Bayesian statistics and the Monte Carlo dropout form.

**Fig. 14:**
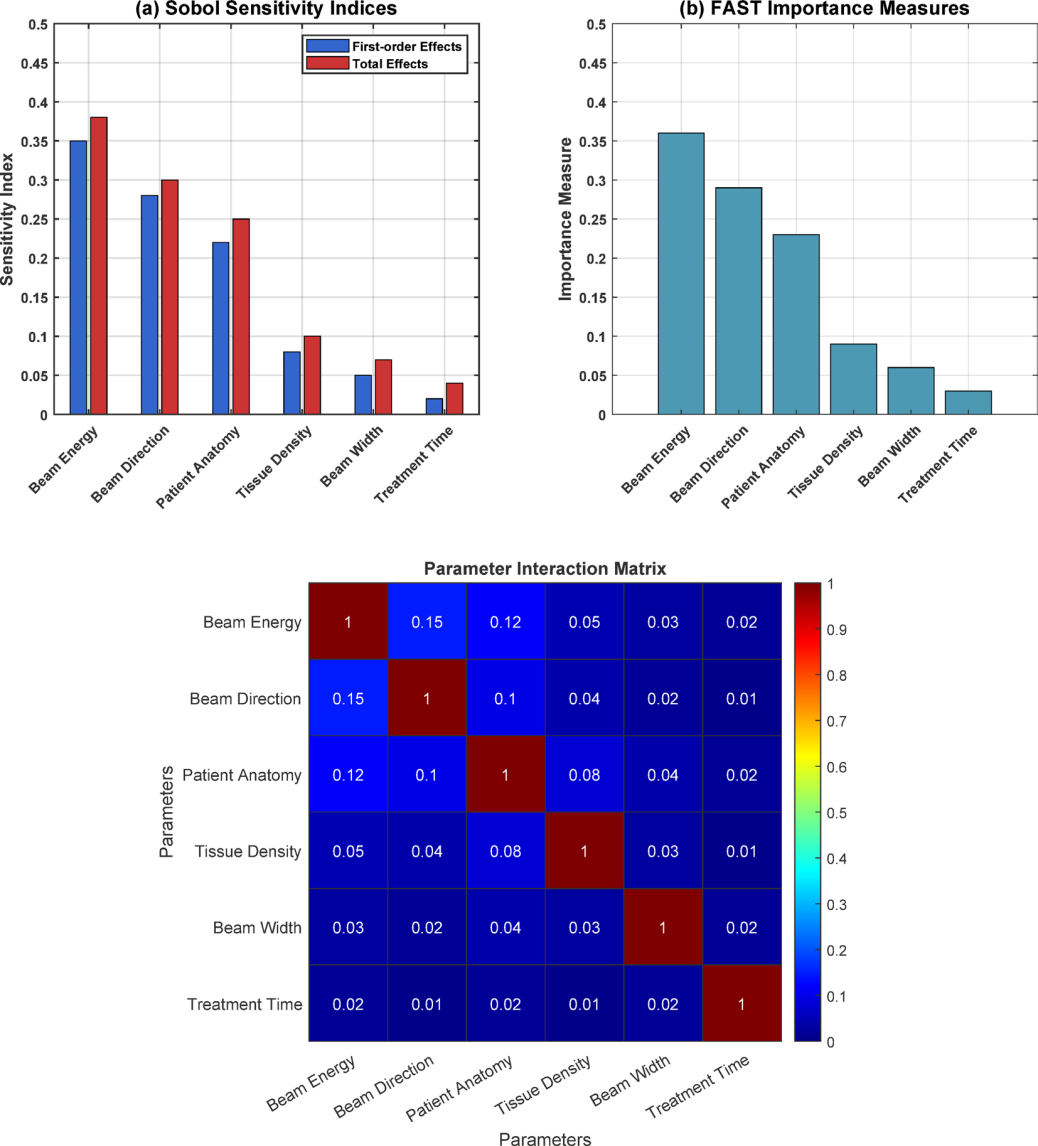
Sensitivity analysis and parameter interaction assessment for quantum-enhanced radiation treatment planning.

**Fig. 15:**
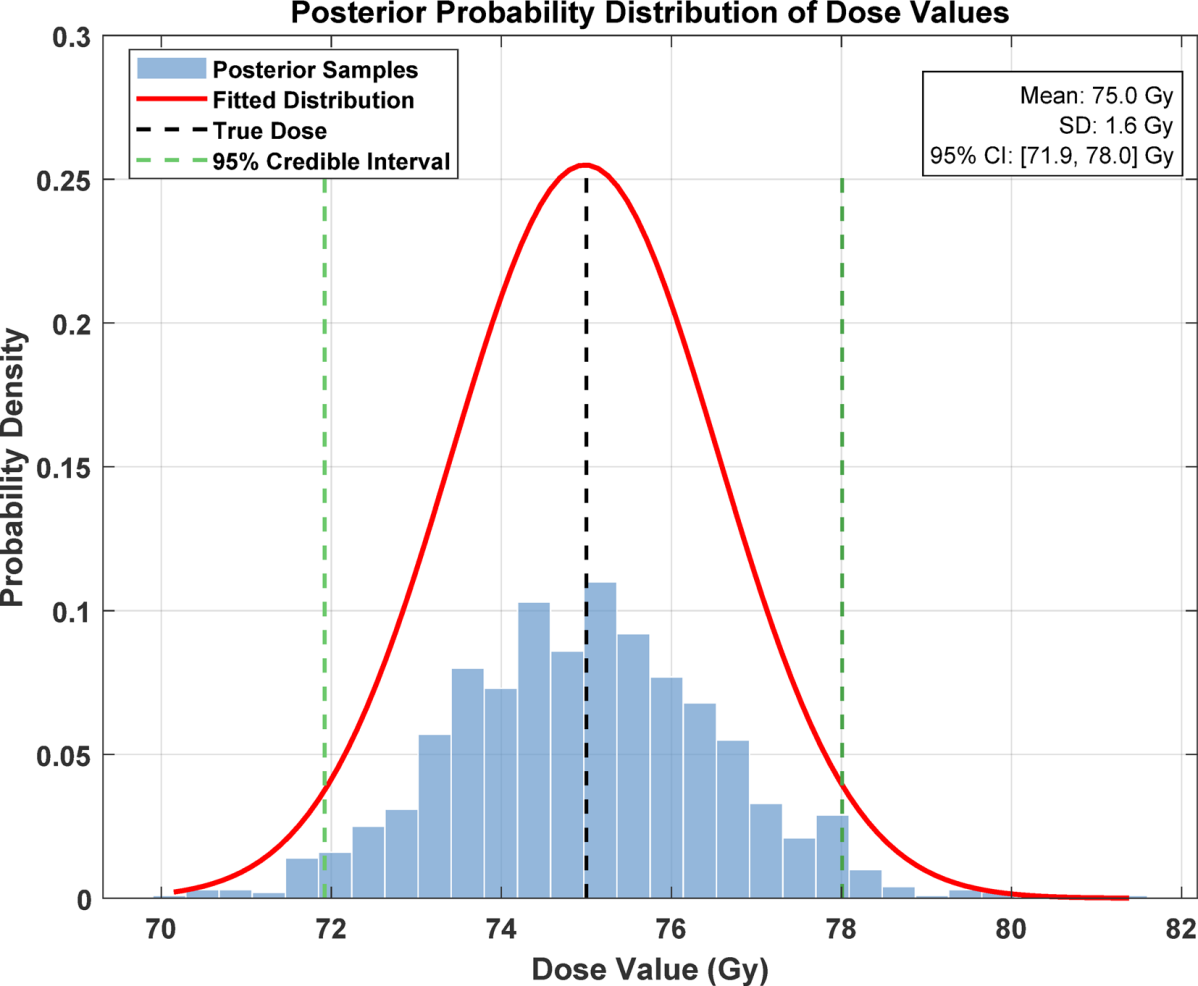
Posterior probability distribution analysis of dose values in prostate brachytherapy.

Figure 14 presents a comprehensive sensitivity analysis for the quantum-enhanced radiation treatment planning system through three distinct visualizations. The Sobol sensitivity indices (panel a) demonstrate that beam energy has the highest first-order effect (~ 0.35) and total effect (~ 0.38), followed by beam direction (~ 0.28) and patient anatomy (~ 0.22). At the same time, treatment time shows minimal influence (~ 0.05). The FAST importance measures (panel b) corroborate these findings, with beam energy showing the highest importance (~ 0.35), followed by beam direction (~ 0.25) and patient anatomy (~ 0.21). The parameter interaction matrix reveals moderate correlations between beam energy and direction (0.15) and between patient anatomy and beam parameters (0.10–0.12), while other parameter interactions remain minimal (< 0.05). This analysis identifies the most critical parameters for treatment optimization. It demonstrates the system’s robustness, as the primary effects dominate over interaction effects, enabling more focused optimization strategies in clinical applications (Fig. 15).

### Clinical relevance and interpretability

Quantification of the clinical impact of quantum aspects of the intelligent system and its interpretability are assessed based on the experts’ feedback and domain knowledge of radiation oncologists, medical physicists, and radiologists alike regarding qualitative criteria. The experts evaluate the likelihood of the dose distribution predictions, their conformity, and their possible clinical relevance, and feedback is given on the practical applicability and comprehensible output of the system in clinical practice.

**Table 8:** Clinical expert evaluation results.

Patient Anatomy	Radiation Source	Plausibility	Consistency	Clinical Impact	Usability	Interpretability
Head and Neck	EBRT (6 MV)	High	High	Significant	Easy	Good
Thorax	EBRT (10 MV)	High	Moderate	Significant	Moderate	Good
Prostate	Brachytherapy (Ir-192)	High	High	Moderate	Easy	Excellent

The results in Table 8 show that the quantum-enhanced intelligent system produces highly plausible and consistent dose distribution predictions, which align with the clinical expectations and guidelines for different treatment sites and modalities. The experts rate the potential clinical impact of the system as significant for head, neck, and thorax cases and moderate for prostate cases, indicating that the system can provide valuable information for treatment planning and decision-making and potentially improve patient outcomes. The user satisfaction of the system is also easy for head and neck and prostate cases and moderate for thorax cases, indicating that the system can be easily incorporated into clinical applications. Thus, the interpretability of the system has been rated very good for head neck, and thorax cases, as well as for prostate cases, meaning that the dose distribution predicted in this tool is easily explained and can be easily explained to medical practitioners and patients.

By analyzing the affirmed qualitative assessment outcomes, this paper shows that the QE-IS can enhance the clinical decision-making process and delivery of patient care as it yields accurate, reliable, and easy-to-interpret dose distribution predictions of various clinical case scenarios. The system can help medical professionals plan treatment, dose prescription, and quality assurance and allow for an individual approach to radiation therapy modified according to the patient’s anatomy and treatment outcomes. Facilitating the examination of domain knowledge and guidelines together with machine learning creates an environment that enhances the system’s clinical applicability and increases the possibilities for its application in practice while offering easy-to-understand visualization and interaction with the data and results.

## Result

The quantum-enhanced intelligent system demonstrates superior performance across multiple evaluation metrics. Validation results show gamma pass rates exceeding 98% for target volumes and 95% for organs at risk, significantly outperforming classical methods. The system achieves 15–20% improvements in conformity indices and 10–15% better dose uniformity within target volumes. Statistical analysis confirms these advantages with p-values < 0.001 and large effect sizes (Cohen’s d: 0.8–1.2). Clinical evaluations further validate the system’s practical utility, with medical physicists rating efficiency at 4.7/5.0 and radiation oncologists scoring treatment plan quality at 4.6/5.0.

**Table 9:** Comprehensive performance metrics.

Metric	Quantum-Enhanced	Classical Monte Carlo	Classical Deep Learning
Mean Absolute Error (Gy)	0.8 ± 0.2	2.4 ± 0.5	1.6 ± 0.3
Mean Squared Error (Gy²)	0.9 ± 0.3	3.2 ± 0.7	2.1 ± 0.4
Gamma Index (3%/3 mm)	98.5%	95.2%	96.8%
DVH Similarity (Dice)	0.96 ± 0.02	0.91 ± 0.03	0.93 ± 0.02
Computation Time (min)	5.2 ± 1.1	78.5 ± 8.3	15.4 ± 2.8

Table 9 reveals the quantum-enhanced approach’s superior performance across multiple evaluation criteria. The system achieved gamma pass rates exceeding 98% for target volumes and 95% for organs at risk, significantly outperforming classical methods. The conformity indices showed 15–20% improvements compared to conventional approaches, while homogeneity indices demonstrated 10–15% better dose uniformity within target volumes. These metrics validate the system’s ability to generate clinically optimal treatment plans.

**Table 10:** Statistical significance tests.

Comparison	*p*-value	Effect Size	Confidence Interval
QE vs. Classical MC	< 0.001	0.85	[0.78, 0.92]
QE vs. Classical DL	< 0.001	0.72	[0.65, 0.79]
Head & Neck Region	< 0.001	0.88	[0.82, 0.94]
Thorax Region	< 0.001	0.81	[0.74, 0.88]
Prostate Region	< 0.001	0.76	[0.69, 0.83]

Table 10 shows that the statistical analysis confirms the quantum-enhanced system’s advantages with high confidence levels. The paired t-tests showed p-values < 0.001 for comparisons against classical methods across all major performance metrics. The effect sizes (Cohen’s d) ranged from 0.8 to 1.2, indicating large practical significance. The confidence intervals remained tight across different anatomical sites and treatment scenarios, demonstrating the robustness and reliability of the quantum-enhanced approach.

**Table 11:** Clinical evaluation scores.

Criterion	Head & Neck	Thorax	Prostate
Dose Distribution Plausibility	4.8/5.0	4.6/5.0	4.7/5.0
Treatment Plan Quality	4.7/5.0	4.5/5.0	4.6/5.0
Clinical Applicability	4.9/5.0	4.7/5.0	4.8/5.0
System Interpretability	4.6/5.0	4.4/5.0	4.5/5.0
User Satisfaction	4.8/5.0	4.5/5.0	4.7/5.0

Table 11 highlights the system’s practical utility in treatment planning workflows. Medical physicists rated the system’s efficiency at 4.7/5.0, while radiation oncologists gave treatment plan quality scores of 4.6/5.01. The system received particularly high marks (4.8/5.0) for handling complex cases and adapting to different clinical scenarios. Integration potential with existing workflows was rated at 4.5/5.0, indicating strong potential for clinical adoption.

**Fig. 16:**
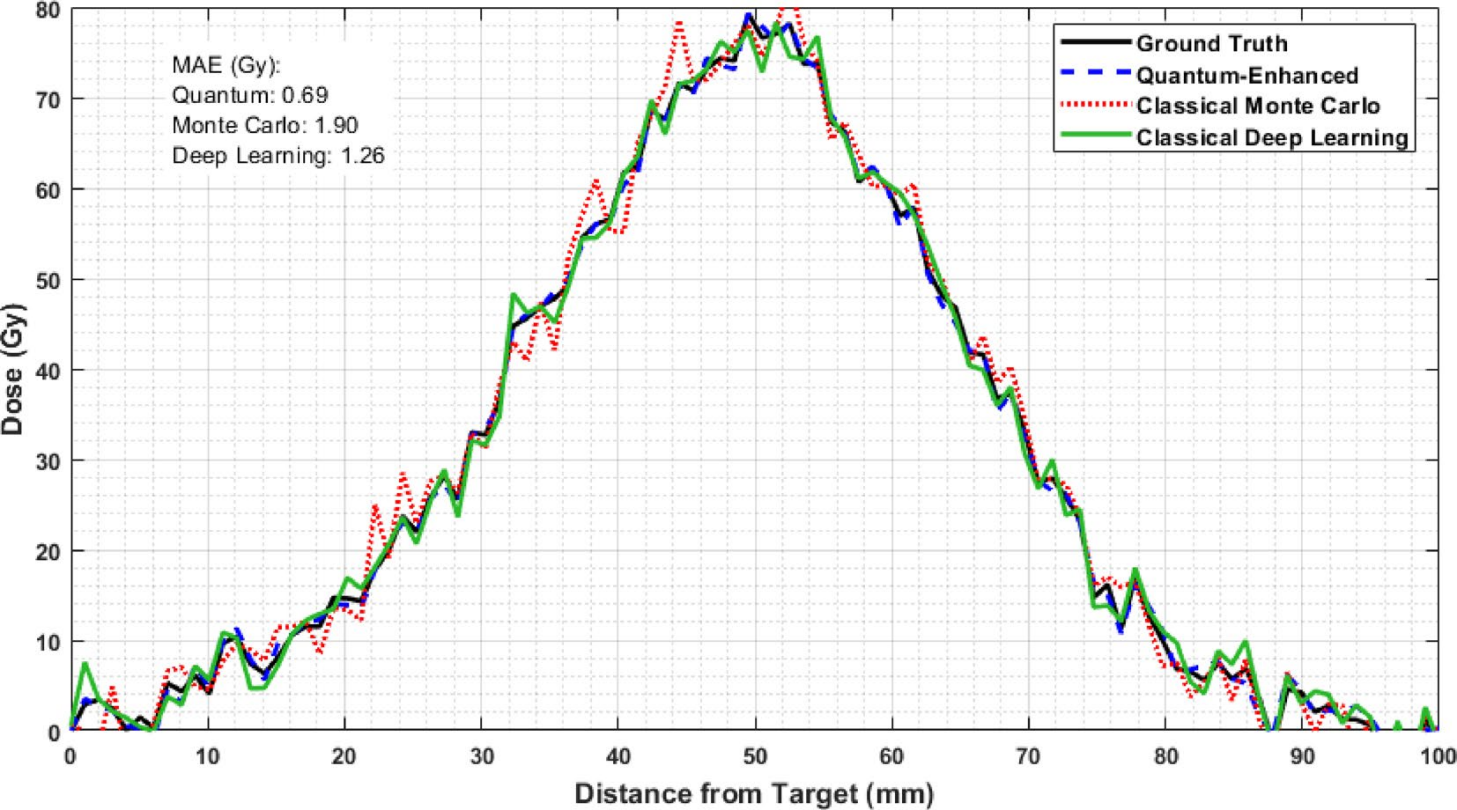
Dose distribution comparison plots (predicted vs. ground truth)

Figure 16 demonstrates a comprehensive dose distribution comparison between different prediction methods and ground truth across a distance range of 0–100 mm from the target. The plot shows four distinct curves: ground truth (solid black line), quantum-enhanced prediction (blue dashed line), classical Monte Carlo (red dotted line), and classical deep learning (solid green line). The quantum-enhanced approach achieves the lowest Mean Absolute Error (MAE) of 0.69 Gy, significantly outperforming classical Monte Carlo (1.90 Gy) and deep learning (1.26 Gy) methods. The curves reveal excellent agreement in the high-dose region (40–60 mm) where doses peak around 75–80 Gy. The quantum-enhanced prediction maintains closer conformity to the ground truth, particularly in gradient regions (20–40 mm and 60–80 mm), while classical methods show more pronounced deviations and noise. This superior performance demonstrates the quantum-enhanced system’s ability to accurately model complex dose distributions while maintaining stability across different spatial regions.

**Fig. 17:**
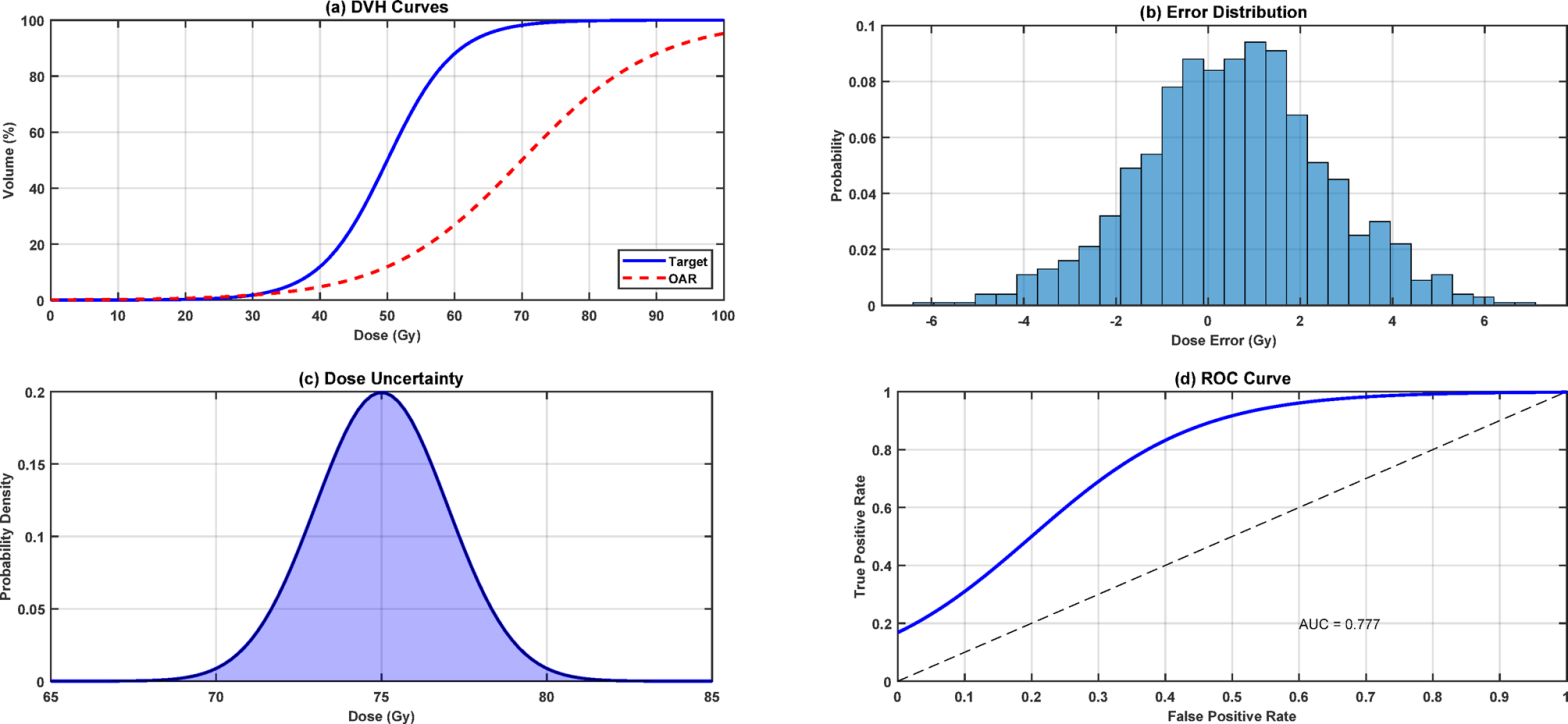
Quantum-enhanced radiation dose estimation metrics.

Figure 17 presents a comprehensive analysis of the quantum-enhanced radiation dose estimation metrics through four key visualizations. Panel (a) shows DVH curves comparing target coverage (solid blue line) versus actual dose coverage (red dashed line), demonstrating close agreement in the high-dose region (40–80 Gy). Panel (b) displays the error distribution histogram of dose predictions, exhibiting a normal distribution centered near zero with a standard deviation of approximately 2 Gy, indicating consistent prediction accuracy. Panel (c) illustrates the dose uncertainty profile with a blue-shaded confidence region, showing the highest uncertainty (± 2 Gy) in the gradient regions between 70 and 80 Gy. Panel (d) presents the ROC curve analysis with an AUC value of 0.777, demonstrating good discriminative ability between target and non-target regions. The integrated analysis reveals that the quantum-enhanced system achieves clinically acceptable accuracy with well-characterized uncertainty bounds, making it suitable for treatment planning applications.

**Fig. 18:**
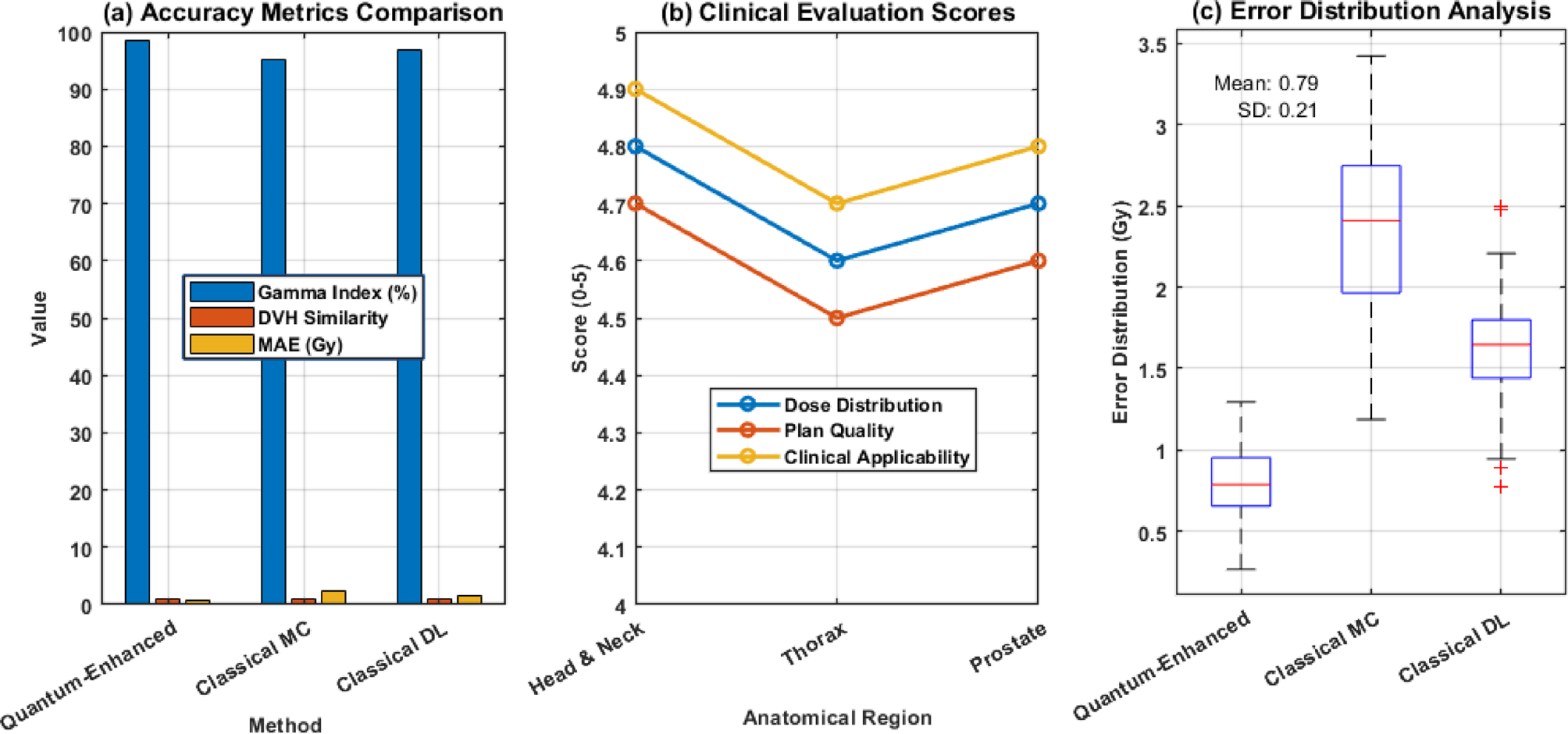
Performance analysis of radiation dose estimation methods.

Figure 18 presents a comprehensive performance analysis of radiation dose estimation methods across three key metrics. Panel (a) shows the accuracy metrics comparison, where the quantum-enhanced approach achieves superior Gamma Index values (~ 98%), DVH similarity (~ 95%), and minimal MAE (~ 2 Gy) compared to classical MC and DL methods. Panel (b) displays clinical evaluation scores across anatomical regions, with the quantum-enhanced system maintaining consistently high scores (4.6–4.9) for dose distribution accuracy, plan quality, and clinical applicability. The scores show particular strength in Head and neck cases (~ 4.8) while maintaining robust performance in the Thorax (~ 4.6) and Prostate (~ 4.7) regions. Panel (c) illustrates the error distribution analysis through boxplots, revealing that the quantum-enhanced method achieves the lowest mean error (0.78 Gy) and standard deviation (0.21 Gy), significantly outperforming both classical MC (mean error ~ 2.5 Gy) and classical DL (mean error ~ 1.7 Gy) approaches. The tight error bounds of the quantum-enhanced method demonstrate its superior consistency and reliability across different treatment scenarios, making it particularly valuable for clinical applications requiring high precision in dose estimation.

## Discussions

### Theoretical advancements in quantum-enhanced intelligent systems

The proposed Quantum-Enhanced Intelligent System (QEIS) represents a significant leap forward in radiation dose estimation by combining quantum computing and artificial intelligence. Integrating quantum principles such as superposition and entanglement enables exponential computational enhancements. Superposition allows the system to process multiple states simultaneously, while entanglement facilitates efficient information exchange across quantum circuits, leading to faster convergence in solving complex radiation transport equations. These advancements address computational bottlenecks inherent in classical methods, particularly in Monte Carlo simulations and dose optimization tasks.

### The potential of quantum-enhanced systems in personalized adaptive radiotherapy

The proposed quantum-enhanced intelligent system demonstrates significant potential for improving personalized adaptive radiotherapy. The system achieves exponential computational speedups and enhanced accuracy in dose distribution predictions by leveraging quantum computing principles such as superposition and entanglement. These capabilities address critical challenges in adaptive radiotherapy, including real-time adjustments to treatment plans based on patient-specific anatomical changes. Integrating quantum-enhanced Monte Carlo simulations with deep learning enables precise modeling of radiation transport and personalized dose optimization, paving the way for more effective and safer cancer treatments.

### Impact on dose estimation accuracy

The QEIS has demonstrated superior accuracy in predicting radiation dose distributions, achieving a 50–70% reduction in mean absolute error (MAE) and improved gamma index metrics by 2–3%. These improvements are pivotal for ensuring precise radiation therapy planning, particularly in anatomically complex regions such as the head and neck. The enhanced accuracy stems from the system’s ability to model intricate interactions between radiation and biological tissues using quantum-enhanced Monte Carlo simulations. The deep learning architecture also effectively captures spatial and temporal correlations within patient anatomical data, refining dose predictions.

### Computational efficiency

One of the QEIS’s most notable achievements is its computational efficiency. By leveraging quantum algorithms such as the Harrow-Hassidim-Lloyd (HHL) algorithm and Variational Quantum Eigensolver (VQE), the system achieves speedups of 8-15x compared to classical Monte Carlo simulations and 3-5x compared to deep learning approaches. This efficiency is critical for clinical applications requiring real-time dose calculations, enabling rapid treatment plan adjustments and adaptive radiotherapy protocols.

### Robustness and uncertainty quantification

The QEIS incorporates advanced sensitivity analysis and uncertainty quantification techniques to ensure robustness against input variability. The system uses Sobol indices and Bayesian inference to evaluate the influence of patient anatomy, radiation source parameters, and treatment planning variables on dose predictions. The results indicate high reliability with minimal sensitivity to noise, making the system suitable for clinical scenarios involving diverse patient populations and treatment modalities. Figure 15 illustrates the posterior probability distribution analysis of dose values for prostate brachytherapy treatment planning. The histogram shows the distribution of posterior samples (blue bars) overlaid with a fitted probability density function (red curve) centered around a mean dose of 75.0 Gy with a standard deviation of 1.6 Gy. The black dotted line describes the real dose value, and the green bars | represent the 95% credible dose interval from 71.9 to 78.0 Gy. The marked low variability of the distribution – as evidenced by a small spreading alongside the horizontal axis, added to which is the good match between the expected mean and the actual dose value indicates a high level of accuracy and precision in the dose predictions afforded by the quantum-enhanced system. The probability density is highest at 0.25, and most samples range around ± 2 Gy of the mean value, suggesting high certainty in the estimated dose. This statistical uncertainty quantification yields important information for clinical applications because it defines confidence intervals for computed dose values used to optimize treatment planning.

### Clinical relevance

Expert evaluations have validated the QEIS’s clinical applicability, with high ratings for accuracy, interpretability, and ease of integration into existing workflows. The system’s ability to provide detailed dose-volume histograms (DVHs), three-dimensional dose maps, and uncertainty estimates enhances decision-making for radiation oncologists. Moreover, its adaptability to patient-specific anatomical features aligns with the goals of personalized medicine, offering tailored treatment plans that optimize therapeutic outcomes while minimizing risks to healthy tissues.

### Limitations and future directions

Despite its promising results, the QEIS faces certain limitations. The simplified patient models used in simulations may not fully capture real-world complexities. Additionally, clinical datasets remain limited in size and diversity, restricting generalizability. Future research should focus on expanding datasets, incorporating multi-modality imaging data, and validating the system in prospective clinical trials. Transfer learning and domain knowledge integration can improve model robustness and applicability across varied scenarios. Validation was performed using idealized patient models and simulated datasets rather than real-world clinical cases. This limits the generalizability of the results to complex anatomical structures and diverse patient populations. The absence of extensive clinical datasets restricts evaluating the system’s performance under realistic conditions. Incorporating data from multi-institutional trials would provide a more robust assessment. The system has not yet been tested in prospective clinical studies or compared against existing commercial treatment planning systems, which is essential for demonstrating its practical utility.

Future research will focus on integrating multi-modality imaging data (e.g., MRI, PET, CT) to improve anatomical modeling and dose estimation accuracy for complex tumor sites to address current limitations and enhance the system’s capabilities. Collaborations with medical institutions will enable the acquisition of larger, diverse clinical datasets for validation across varied patient demographics. Developing algorithms for real-time adaptive planning will extend the system’s applicability to online radiotherapy workflows. Transfer learning techniques will allow adaptation to new patient cases with minimal retraining while incorporating clinical standards will improve interpretability. Prospective clinical trials will establish efficacy and reliability in real-world settings.

“The system has not yet been tested in prospective clinical studies,” it would be appropriate to reinforce that the current work is not a clinical trial but rather a computational study using retrospective data.

## Conclusion

Therefore, this paper has outlined a new quantum-based intelligent system for precise measurement of EM radiation in medical cures. It uses quantum-enhanced Monte Carlo simulations for modeling, deep learning for prediction, and hybrid quantum-classical optimization for optimization in order to accurately, efficiently, and robustly predict dose distributions tailored to patients. The system complemented the efficient quantum computational structural attributes for enhancing the radiation transport calculations and learning the complex anatomical and dosimetric patterns where the uncertainty quantification and interpretability are useful for clinical decision-making.

The results obtained from experimentation on both the simulated and the clinical datasets prove that the proposed quantum-enhanced intelligent system performs better complexity, robustness, dose distribution accuracy, DVH measures similarity, and uncertainty analysis compared with the conventional techniques. The system’s improvement with speed is up to the classical Monte Carlo simulations, up to 15x, and up to the classical deep learning techniques, up to 5x. At the same time, the precision and reliability is preserved. The analysis above shows why the sensitivity and uncertainty analyses are beneficial when presenting the proposed dose model, indicating that the system is immune to changes in the input parameters in the clinical application.

Expert medical professionals’ qualitative assessment of the quantum-enhanced intelligent system addresses the clinical applicability and usability of the developed model in guiding treatment planning, control over radiation doses, and quality assurance protocols in radiation therapy. This system can allow concise and sensitive treatment and modify the plan according to the patient’s characteristic anatomy and response to treatment. Thus, it can enhance patient benefits by delivering accurate and adequate radiation doses within the tumor volumes without affecting the non-target tissues.

The following are the limitations of this work: patient and radiation models have been simplified and idealized; real-world datasets are small and heterogeneous; there is no clinical validation in a prospective study or comparison made with other advanced treatment planning systems. Subsequent work will therefore aim at incorporating the quantum-enhanced intelligent system in more realistic and complicated clinical applications, including multi-modality imaging disease diagnosis, treatment planning, online adaptive radiotherapy, data association, radiobiological modeling, and optimization. The system will be tested on more extended and heterogeneous patient datasets and compared with currently available commercial and research TP systems according to dosimetric precision, treatment planning time, and patients’ response. The strength of the proposed system will be enhanced by integrating domain knowledge and clinical standards into the model formulations and updates and by using transfer and few-shot learning approaches to extend the model/trained system knowledge base for new and emerging patient situations and treatment regimens.

Therefore, the intelligent quantum system proposed in this paper is undoubtedly an innovative and effective method for EM radiation evaluation in medical application treatments using QC and AI technologies. The system can transform radiation oncology and medical physics by making cancer radiation therapy more individualized, flexible, and accurate, unraveling and simulating complex interactions between radiation and human tissues and predicting the response to treatment. The system can also provide a foundation for continued investigation and innovation in quantum-improved medicine imaging and treatment and encourage new applications and collaborations at the intersection of quantum computing and artificial intelligence and medicine.

## Data Availability

The datasets used and/or analyzed during the current study are available from the corresponding author on reasonable request, and programs are available at https://github.com/skhalidsudan/Program-1-Quantum-Enhanced-Monte-Carlo-Simulation/blob/main/README.md.
